# Mesothelial‐to‐mesenchymal transition as a possible therapeutic target in peritoneal metastasis of ovarian cancer

**DOI:** 10.1002/path.4889

**Published:** 2017-04-03

**Authors:** Angela Rynne‐Vidal, Chi Lam Au‐Yeung, José A Jiménez‐Heffernan, María Luisa Pérez‐Lozano, Lucía Cremades‐Jimeno, Carmen Bárcena, Ignacio Cristóbal‐García, Concepción Fernández‐Chacón, Tsz Lun Yeung, Samuel C Mok, Pilar Sandoval, Manuel López‐Cabrera

**Affiliations:** ^1^ Centro de Biología Molecular‐Severo Ochoa (CBMSO)Departamento de Biología Celular e InmunologíaMadridSpain; ^2^ Department of Gynecologic Oncology and Reproductive MedicineThe University of Texas MD Anderson Cancer CenterHoustonTX 77030USA; ^3^ Departamento de Anatomía Patológica, Hospital Universitario La PrincesaInstituto de Investigación Sanitaria Princesa (IP)MadridSpain; ^4^ Departamento de Anatomía PatológicaHospital Universitario 12 de OctubreMadridSpain; ^5^ Servicio de Oncología GinecológicaHospital de la Zarzuela SanitasMadridSpain

**Keywords:** peritoneal metastasis, ovarian cancer, carcinoma‐associated fibroblasts, mesothelial‐to‐mesenchymal transition, ascites

## Abstract

Peritoneal dissemination is the primary metastatic route of ovarian cancer (OvCa), and is often accompanied by the accumulation of ascitic fluid. The peritoneal cavity is lined by mesothelial cells (MCs), which can be converted into carcinoma‐associated fibroblasts (CAFs) through mesothelial‐to‐mesenchymal transition (MMT). Here, we demonstrate that MCs isolated from ascitic fluid (AFMCs) of OvCa patients with peritoneal implants also undergo MMT and promote subcutaneous tumour growth in mice. RNA sequencing of AFMCs revealed that MMT‐related pathways – including transforming growth factor (TGF)‐β signalling – are differentially regulated, and a gene signature was verified in peritoneal implants from OvCa patients. In a mouse model, pre‐induction of MMT resulted in increased peritoneal tumour growth, whereas interfering with the TGF‐β receptor reduced metastasis. MC‐derived CAFs showed activation of Smad‐dependent TGF‐β signalling, which was disrupted in OvCa cells, despite their elevated TGF‐β production. Accordingly, targeting Smad‐dependent signalling in the peritoneal pre‐metastatic niche in mice reduced tumour colonization, suggesting that Smad‐dependent MMT could be crucial in peritoneal carcinomatosis. Together, these results indicate that bidirectional communication between OvCa cells and MC‐derived CAFs, via TGF‐β‐mediated MMT, seems to be crucial to form a suitable metastatic niche. We suggest MMT as a possible target for therapeutic intervention and a potential source of biomarkers for improving OvCa diagnosis and/or prognosis. © 2017 The Authors. *The Journal of Pathology* published by John Wiley & Sons Ltd on behalf of Pathological Society of Great Britain and Ireland.

## Introduction

A common characteristic of cancers that progress with peritoneal metastasis is that they evolve very rapidly, without symptoms, and are diagnosed at advanced stages 1. Debulking surgery followed by platinum–taxane chemotherapy is the current standard of treatment, and improves survival rates in selected patients 2; however, there is still limited scope for curing peritoneal carcinomatosis. In particular, survival rates of patients with ovarian cancer (OvCa) at advanced stages are 10–30% 3, making it the fifth leading cause of cancer death in women 4.

The peritoneum is composed of a monolayer of mesothelial cells (MCs) that lines a connective tissue, consisting of few fibroblasts, adipocytes, immune cells, and vessels 5. We have previously shown that a subset of carcinoma‐associated fibroblasts (CAFs) in peritoneal metastases are derived from MCs via mesothelial‐to‐mesenchymal transition (MMT) 6, 7, which is an epithelial‐to‐mesenchymal transition (EMT)‐like process 8, 9. During MMT, MCs acquire a fibroblast‐like phenotype, with increased capacity to migrate and to invade the submesothelial compact zone. The acquisition of mesenchymal features by MCs results from a profound genetic reprogramming 8, 9.

CAFs are activated fibroblasts integrated in the tumour architecture that favour cancer cell survival, proliferation, and invasion. They synthesize an array of extracellular matrix components (ECM), cytokines and growth factors that contribute to the transformation of the tumour niche and also promote angiogenesis 10. However, the role of MC‐derived CAFs in the peritoneal tumour stroma has not been studied in depth.

At early stages, peritoneal metastasis develops as a consequence of the accumulation of alterations in cancer cells and a reversible mesenchymal conversion of these cells via EMT, enabling them to detach from the primary tumour into the peritoneal cavity 1. However, in the establishment of metastasis, the metastatic niche is as important as the intrinsic features of the tumour 11. Complex bidirectional interactions between metastatic cancer cells and the peritoneal environment seem to be crucial for colonization of the peritoneum, and MMT has been recently reported to play an important role in the processes of attaching to and invading through the peritoneal membrane 6, 12, 13.

Tumours that arise in the peritoneal cavity, most notably OvCa, often progress with an accumulation of ascitic fluid 1. Many cytokines and growth factors are present in OvCa ascitic fluid 14. In this regard, transforming growth factor (TGF)‐β is frequently found in ascites 15, and is also a major inducer of MMT 9. Herein, we characterize ascitic fluid‐isolated MCs (AFMCs) from OvCa patients with peritoneal metastasis in order to investigate whether they undergo MMT.

## Materials and methods

### Culture and treatments of MCs and OvCa cell line

Human peritoneal MCs (HPMCs) were isolated by trypsinization of omentum samples obtained from non‐oncological patients undergoing abdominal surgery 8. AFMCs were obtained by centrifuging (500 g, 5 min.) peritoneal effusions of patients with International Federation of Gynecology and Obstetrics Stage III ovarian serous carcinoma. MCs were grown in Earle's M199 medium, supplemented with 20% fetal bovine serum (FBS) and 2% Biogro‐2 (Biological Industries, Beit Haemek, Israel). The purity of the cultures was determined by flow cytometry and/or immunofluorescence for standard mesothelial markers, intercellular adhesion molecule‐1 and calretinin, and by ruling out any contamination with endothelial cells or macrophages by finding cultures to be negative for CD31 and CD45.

To induce MMT *in vitro*, HPMCs were treated with 0.5 ng/ml TGF‐β1 (R&D Systems, Minneapolis, MN, USA) plus 2.5 ng/ml interleukin (IL)‐1β (R&D Systems) (T + I) for 72 h 8.

The human ovarian carcinoma cell line SKOV3 expressing luciferase (SKOV3‐luc‐D3) (Caliper Life Sciences, Hopkinton, MA, USA) was cultured in McCoy's 5A medium supplemented with 10% FBS, with geneticin used as a selection agent.

In additional experiments, HPMCs and SKOV3 cells were stimulated with TGF‐β1 (4 ng/ml; R&D Systems) for 1 or 6 h.

### Animal experiments

All experiments were performed with Swiss nu/nu 6–7‐week‐old female mice (Charles River Laboratories, Barcelona, Spain). The experimental protocols conformed to the National Institutes of Health Guide for Care and Use of Laboratory Animals, and were approved by the Animal Ethics Committee of the Unidad de Experimentación Animal of Centro de Biología Molecular Severo Ochoa (CBMSO) (Madrid, Spain).

#### Subcutaneous xenograft mouse model

In a preliminary assay, 1 × 10^6^ SKOV3‐luc‐D3 cells were inoculated into the left flank of mice, or co‐inoculated into the right flank with 0.5 × 10^6^ AFMCs, and tumour‐produced bioluminescence was monitored for 4 weeks. In additional experiments, mice were co‐inoculated in the left flank with a combination of 1 × 10^6^ SKOV3‐luc‐D3 cells and 0.5 × 10^6^ HPMCs. On the right flank, mice were co‐inoculated with 1 × 10^6^ SKOV3‐luc‐D3 cells and either 0.5 × 10^6^ HPMCs T + I or 0.5 × 10^6^ AFMCs. Luciferase signals were monitored for 5 weeks.

#### Mouse model of carcinoma peritoneal dissemination, pre‐conditioning deliveries, and treatments

To pre‐condition the peritoneum, TGF‐β1 expression was induced in the peritoneal cavity with adenoviral vectors. HEK 293‐A cells were infected with a control adenovirus or with an adenovirus encoding active TGF‐β1, kindly provided by F. Rodríguez‐Pascual (CBMSO, Madrid, Spain) 16. Four days post‐infection, adenoviral particles were re‐collected, purified with an Adeno‐X Maxi Purification Kit (Clontech Laboratories, Mountain View, CA, USA), and titrated with an Adeno‐X Rapid Titer Kit (Clontech). Mice were infected with 1 × 10^7^ infection‐forming units (IFUs) of control or TGF‐β1‐encoding adenovirus. Seven days post‐infection, mice were intraperitoneally inoculated with 5 × 10^6^ SKOV3‐luc‐D3 cells, and tumour‐produced bioluminescence signals were monitored twice weekly for 6 weeks.

In an additional approach to induce MMT, conditioned medium from SKOV3 cells (maintained in 1% FBS/McCoy's 5A medium for 48 h) was centrifuged (500 X_*g*_, 5 min.) and administered intraperitoneally to mice (*n* = 2). Two days later, mice were killed and peritoneal tissue samples were fixed for immunohistochemical staining. To assess the effects of interference with TGF‐β1 signalling, a total of 24 mice were treated with either a TGF‐β receptor I inhibitor (GW788388) (3 mg/kg per day) (Tocris Bioscience, Bristol, UK) or the vehicle dimethyl sulphoxide. Two days later, six mice from each group received intraperitoneal administration of either SKOV3‐conditioned medium or control medium plus a repeat dose of TGF‐β receptor I inhibitor. Then, mice were inoculated with 5 × 10^6^ SKOV3‐luc‐D3 cells, and tumour growth was monitored by bioluminescence imaging for 6 weeks.

In a preliminary assay, mice were intraperitoneally inoculated with either phosphate‐buffered saline (PBS), 1 × 10^9^ IFUs of control lentivirus, or 1 × 10^9^ IFUs of Smad3 shRNA lentivirus, and killed 4 days later. Smad3 knockdown was verified in peritoneal tissue samples by western blotting. Then, a total of 18 mice were randomly grouped to be intraperitoneally infected with 1 × 10^9^ IFUs of either control or Smad3 shRNA‐producing lentiviral particles. Four days post‐infection, mice were inoculated intraperitoneally with 5 × 10^6^ SKOV3‐luc‐D3 cells, and luciferase signals were monitored for 6 weeks.

### RNA sequencing and data analysis

Control HPMC and trans‐differentiated AFMC samples were lysed in TRI Reagent (Ambion, Austin, TX, USA) to obtain total RNA. RNA integrity was checked with the Agilent Bioanalyzer 2100 (Agilent Technologies, Santa Clara, CA, USA). Samples were depleted of rRNA, and RNA was then sheared into smaller fragments with a Covaris S220 (Covaris, Woburn, MA, USA). The cDNA library was prepared with the Beckman SPRIworks system (Beckman Coulter, Fullerton, CA, USA). Library fragments hybridize to complementary oligonucleotides, and clusters of clones were generated in the cBOT instrument (Illumina, San Diego, CA, USA). Libraries were sequenced with a HiSeq 2000 (Illumina). Data files from transcriptome profiling analysis were deposited in the Gene Expression Omnibus (GEO) repository and assigned the GEO accession number GSE84829. Further details can be found in supplementary material, Supplementary materials and methods.

### Patient samples

A total of eight ascites samples from different patients were studied: five were analysed for MMT‐related markers, and three were used for RNA sequencing. In addition, peritoneal metastases from 11 serous ovarian carcinomas and two colon cancers were used for immunohistochemical staining. Informed written consent was obtained from the patients, with the approval of the Ethics Committee of Hospital de la Princesa (Madrid, Spain), Hospital de la Zarzuela (Madrid, Spain), Hospital 12 de Octubre (Madrid, Spain) and MD Anderson Cancer Center (Houston, TX, USA). These studies conformed to the Declaration of Helsinki, and were approved by the Ethics Committee of CBMSO (Madrid, Spain).

Procedures for *in vivo* bioluminescence imaging, quantitative reverse transcription polymerase chain reaction (RT‐qPCR), immunofluorescence, immunohistochemistry, western blotting, lentiviral production and statistics are described in supplementary material, Supplementary materials and methods. Specific human primers for RT‐qPCR are shown in supplementary material, Table S1.

## Results

### AFMCs undergo MMT ex vivo and promote the growth of OvCa cells in a subcutaneous xenograft mouse model

**Figure 1:**
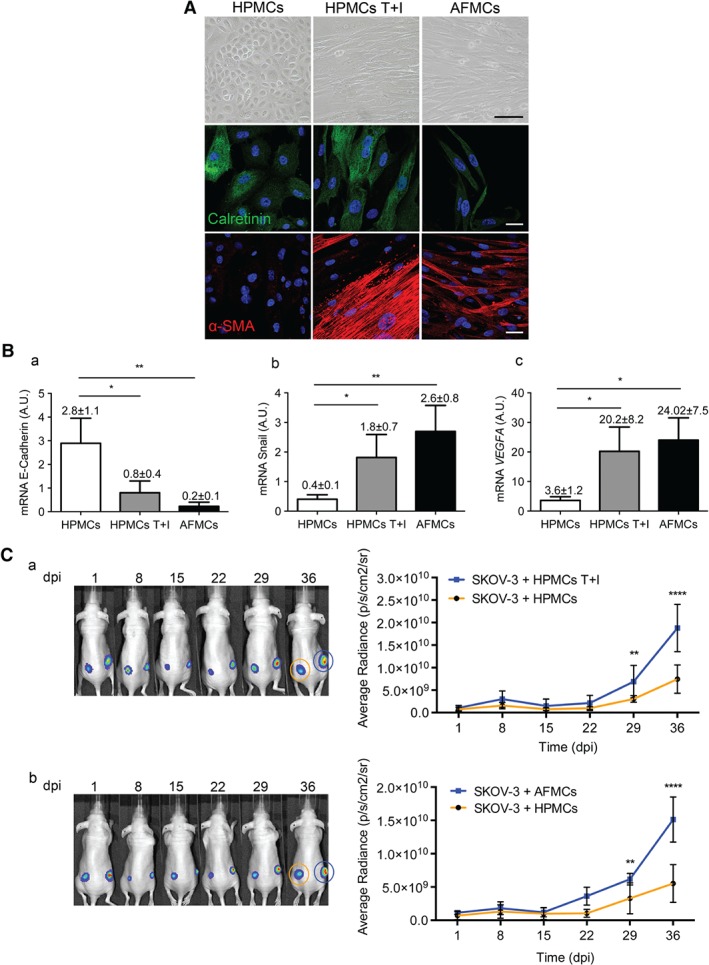
AFMCs undergo MMT ex vivo and favour tumour progression in a subcutaneous xenograft mouse model. (A) Representative microscopy images of HPMCs, HPMCs treated with TGF‐β1 plus IL‐1β (T + I), and AFMCs in culture. Under phase contrast, the altered morphology of AFMCs is similar to that observed in HPMCs T + I (scale bar: 100 µm). Immunofluorescence staining for calretinin (green) confirms the MC nature, and positive α‐SMA (red) expression indicates AFMC conversion into myofibroblasts. [4′,6‐Diamidino‐2‐phenylindole: blue. Scale bars: 25 µm.] (B) Transcript levels of MMT markers, analysed by RT‐qPCR in HPMCs (n = 8), HPMCs T + I (n = 8), and AFMCs (n = 5). E‐cadherin expression is repressed and, conversely, the expression of Snail and VEGF is induced in AFMCs and HPMCs T + I as compared with control HPMCs. Bar graphics represent mean ± standard error of the mean (SEM). Symbols represent the statistical differences between groups (*p ≤ 0.05; **p ≤ 0.005). A.U., absolute units. (C) SKOV3‐luc‐D3 cells were co‐inoculated with control HPMCs into the left flank of mice, and with either HPMCs T + I (n = 7) (a) or AFMC‐derived myofibroblasts (n = 5) (b) in the right flank. Mice of both groups were monitored for 36 days. Representative bioluminescence images show the subcutaneous growth of SKOV3‐luc‐D3 cells plus HPMCs (orange circle) (a, b) as compared with SKOV3‐luc‐D3 cells plus HPMCs T + I (a, blue circle) or SKOV3‐luc‐D3 cells plus trans‐differentiated AFMCs (b, blue circle), over the duration of the experiment. Quantification of bioluminescence showed that tumour growth was increased in the right flank, where MCs that had undergone MMT had been used (a, b). Graphs represent mean average radiance (expressed as photons/s/cm^2^/sr) of SKOV3‐luc‐D3 cells ± SEM. Symbols represent the statistical differences between groups (**p ≤ 0.01; ****p ≤ 0.0001). dpi, days post‐inoculation.

AFMCs from OvCa patients with peritoneal implants cultured *ex vivo* had a fibroblast‐like morphology, with a similar appearance to that of HPMCs trans‐differentiated *in vitro*. Positive immunofluorescence staining for calretinin confirmed their MC nature, and α‐smooth muscle actin (α‐SMA) staining indicated that AFMCs had been converted to myofibroblasts (Figure 1A). To verify the mesenchymal conversion of AFMCs, conventional MMT‐related marker expression was quantified by RT‐qPCR. E‐cadherin expression was significantly repressed in AFMCs, and, conversely, Snail and vascular endothelial growth factor (VEGF) were upregulated as compared with control HPMCs (Figure 1B).

To study the role of trans‐differentiated AFMCs in tumour growth, a subcutaneous xenograft mouse model was used. Although SKOV3‐luc‐D3 cells are efficient in establishing peritoneal metastases, a preliminary assay showed that cancer cells alone are unable to grow when inoculated subcutaneously (supplementary material, Figure S1). The differential behaviour of cancer cells in these two microenvironments suggests that MC‐derived CAFs may be key players in peritoneal metastasis. Therefore, SKOV3‐luc‐D3 cells were subcutaneously co‐inoculated with control HPMCs into the left flank, and with either *in vitro* or *ex vivo* trans‐differentiated MCs in the right flank. In both cases, tumour growth was significantly increased in the right flank, where MCs that had undergone MMT had been co‐injected (Figure 1C).

### Identification of an MMT gene signature in AFMCs

**Figure 2:**
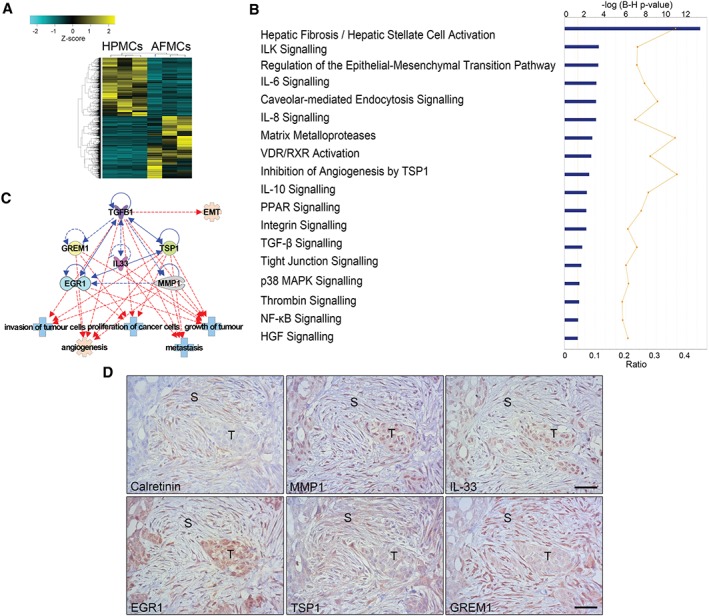
RNA sequencing analysis of AFMCs and protein validation in peritoneal metastasis biopsies. (A) Heat‐map representing the differentially expressed genes in control HPMCs (n = 3) and trans‐differentiated AFMCs (n = 3). (B) Significantly differentially regulated canonical pathways analysed with IPA software. The y‐axis indicates the statistical significance, calculated by use of the Benjamini–Hochberg correction [−log(P‐value) = 1.3]. The yellow threshold line represents this cut‐off. (C) Interactions between five molecules selected from upregulated genes in the dataset (MMP1, IL33, EGR1, TSP1, and GREM1) with TGF‐β1 (blue lines) and tumour‐related functions (red lines). Continuous lines represent direct relationships. Dotted lines represent indirect interactions. (D) A peritoneal implant of an OvCa biopsy reveals the presence of spindle‐like cells surrounding tumour micronodules, stained for calretinin, to indicate their mesothelial origin. Serial sections of the same case show marked staining for MMP1, IL‐33, EGR1, TSP1 and GREM1 overlapping with stromal areas where mesothelial‐derived fibroblastic cells accumulate. The same markers are also detected with variable intensity within the tumour parenchyma. S, stroma; T, tumour. Scale bars: 50 µm.

RNA sequencing analysis was carried out on AFMCs from OvCa patients in comparison with control HPMCs. Expression data for each gene within each sample were used to create a heat‐map for cluster classification, which revealed a clear separation between HPMCs and AFMCs (Figure 2A). Ensembl ID of differentially expressed genes, i.e. those with a *q*‐value of ≤0.05 and at least a twofold change in expression, were submitted to analysis with Ingenuity Pathway Analysis (IPA) software. The analysis revealed 1997 genes that were upregulated and 1646 genes that were downregulated in AFMCs as compared with HPMCs. A summary of the top 100 upregulated and top 100 downregulated genes in AFMCs is shown in supplementary material, Tables S2 and S3. Among the canonical pathways that were significantly differentially regulated, many were related to MMT/EMT and/or OvCa progression (Figure 2B). Upstream regulators represented in our dataset were also identified, and, among the top upregulated ones, we found five inducers of MMT/EMT: tumour necrosis factor (TNF)‐α, TGF‐β1, IL‐1β, hepatocyte growth factor (HGF), and IL‐6 (supplementary material, Table S4).

To validate the results from the RNA sequencing analysis, we selected five upregulated genes: three from the top 100 [encoding matrix metalloproteinase (MMP) 1, IL‐33, and early growth response 1 (EGR1)], and two with roles in regulating the TGF‐β pathway [encoding thrombospondin 1 (TSP1) and gremlin 1 (GREM1)] 17, 18. The interaction between these molecules and with TGF‐β1 suggests possible interesting roles in peritoneal metastasis‐related processes, including invasion, proliferation and growth of tumour cells, and angiogenesis (Figure 2C). Immunohistochemical staining of these proteins in biopsies from human OvCa peritoneal implants showed mesothelial‐derived (calretinin‐positive) spindle‐like cells in the stroma tissue surrounding tumour nodules, overlapping with areas with marked staining for MMP1, IL‐33, EGR1, TSP1, and GREM1. Adjacent tumour cells showed no staining or variable intensity patterns for the same markers (Figure 2D). Moreover, in the mesothelial surface (calretinin‐positive) from the same biopsies, staining was intense for MMP1, EGR1, and GREM1, and variable for IL‐33 and TSP1 (supplementary material, Figure S2).

### MMT via TGF‐β1 in the peritoneum renders it more susceptible to metastasis

**Figure 3:**
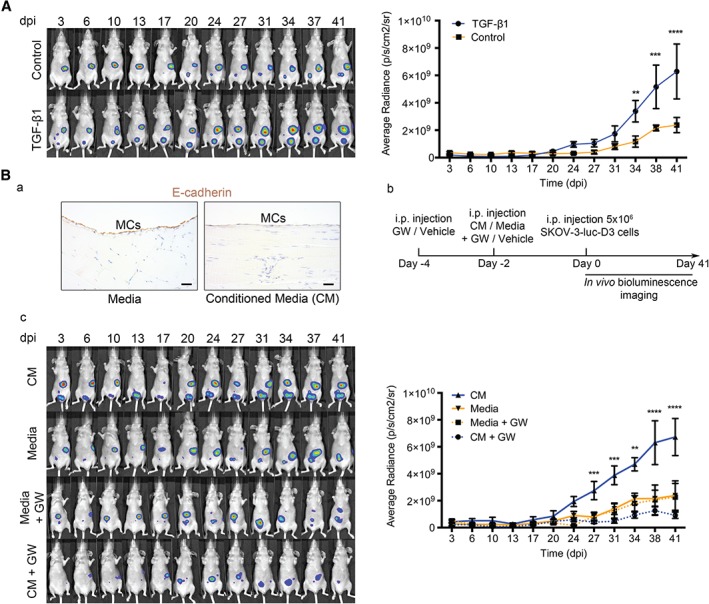
OvCa‐secreted TGF‐β transforms the pre‐metastatic peritoneum, favouring tumour progression. (A) Representative images of in vivo monitoring of SKOV3‐luc‐D3 cells in mice pre‐conditioned with TGF‐β1‐encoding adenovirus or control. Quantification of bioluminescence showed that tumour growth was increased in mice pre‐conditioned with TGF‐β1 adenovirus (n = 6) as compared with control adenoviral pretreatment (n = 6). (B) (a) Representative images of E‐cadherin immunostaining in the mesothelial monolayer of a mouse killed 2 days after being pre‐conditioned with conditioned medium (CM) from OvCa cells or control medium. Scale bars: 25 µm. (b) Diagram of experimental design. (c) Representative images of in vivo monitoring of SKOV3‐luc‐D3 cells and quantification of bioluminescence showed that intraperitoneal tumour growth was higher in mice pretreated with SKOV3 medium (CM). The TGF‐β receptor I inhibitor (GW) reduced tumour growth to levels comparable to those of mice whose peritoneums had not been pre‐conditioned (control medium). n = 6 per group. All mice were monitored for 41 days. Graphs represent mean average radiance (expressed as photons/s/cm^2^/sr) of SKOV3‐luc‐D3 cells ± standard error of the mean. Symbols represent the statistical differences over time between groups (**p ≤ 0.01; ***p ≤ 0.001; ****p ≤ 0.0001). dpi, days post‐inoculation; i.p., intraperitoneal.

It has been shown that MCs that have undergone MMT promote increased attachment and invasion by cancer cells 6, 13. On the basis of these observations, we hypothesized that a peritoneum in which MMT had taken place could be more receptive to metastasis. Given that TGF‐β1 is a key MMT inducer 16 and also appeared as a key regulator in the RNA sequencing data, we were interested in studying its role in peritoneal tumour progression. Thus, mouse peritoneum was pre‐conditioned by overexpression of TGF‐β1 with adenoviral delivery, followed by SKOV3‐luc‐D3 cell intraperitoneal inoculation. Tumour growth was significantly higher in mice whose peritoneums had been pretreated with TGF‐β1 than in those in which a control adenovirus had been used (Figure 3A).

We have previously reported that conditioned media from OvCa cell cultures have a high concentration of TGF‐β1 and induce MMT *in vitro* 6. Here, we observed that pre‐conditioning the peritoneum of mice for 2 days with conditioned medium from SKOV3 cells decreased E‐cadherin expression in the mesothelial monolayer, indicating that an early MMT had taken place (Figure 3Ba). To study the role of TGF‐β1 accumulated in OvCa ascitic fluid, mice were pretreated with a TGF‐β1 receptor I inhibitor (GW788388) and then with conditioned medium from SKOV3 cells, and this was followed by SKOV3‐luc‐D3 intraperitoneal delivery (Figure 3Bb). Tumour growth was significantly higher in mice in which an early MMT had been induced with cancer cell medium. When TGF‐β1 receptor I was inhibited and cancer cell medium was used, tumour growth was reduced to levels comparable to those of mice whose peritoneums had not been pre‐conditioned (Figure 3Bc).

### Crosstalk between mesothelial‐derived CAFs and OvCa cells takes place via the TGF‐β1–pSmad3 pathway

**Figure 4:**
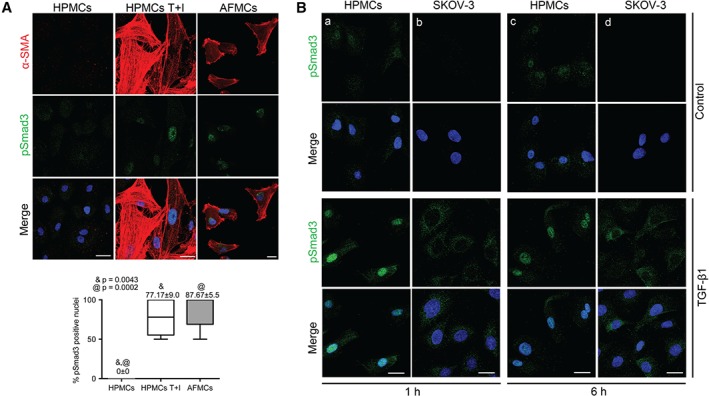
The TGF‐β1–Smad3 pathway is activated in AFMCs, and truncated in OvCa cells. (A) Double immunofluorescence staining for α‐SMA (red) and pSmad3 (green) in HPMCs T + I and AFMCs indicates Smad3‐dependent TGF‐β1 pathway activation in both in vitro and ex vivo trans‐differentiated MCs as compared with double‐negative control cells. Scale bars: 25 µm. pSmad3‐positive nuclei were quantified; the box plot represents mean ± standard error of the mean. Symbols represent the statistical differences between groups. (B) Treatment of HPMCs and SKOV3 cells with TGF‐β1 for 1 and 6 h. Immunofluorescence images show that, upon TGF‐β1 treatment, pSmad3 translocates to the nucleus in HPMCs (a, c) and remains cytoplasmic in OvCa cells (b, d). 4′,6‐Diamidino‐2‐phenylindole: blue. Scale bars: 25 µm.

It is well established that TGF‐β1 induces MMT through both Smad‐dependent and Smad‐independent pathways 9, 19. Smad3 is an important downstream mediator in the Smad‐dependent signalling of TGF‐β1; once phosphorylated, pSmad3 translocates into the nucleus and regulates gene transcription 9. However, the response to TGF‐β1 through Smad3 has not been analysed in MC‐derived CAFs. Double immunofluorescence staining for α‐SMA and pSmad3 showed that control MCs were negative for both markers, whereas a significant increase in the nuclear expression of pSmad3 was observed in both *in vitro* and *ex vivo* trans‐differentiated (α‐SMA positive) MCs (Figure 4A).

Analysis of the response of OvCa cells and HPMCs to TGF‐β *in vitro* for 1 and 6 h showed that pSmad3 was translocated to the nucleus in HPMCs, whereas it remained cytoplasmic in OvCa cells, suggesting that the TGF‐β1–Smad3 pathway is truncated in these tumour cells (Figure 4B).

Accordingly, we analysed pSmad3 localization in the peritoneums of tumour‐bearing mice, where CAFs (α‐SMA‐positive) expressing nuclear pSmad3 were observed in the tumour stroma and, similarly to what was shown by our *in vitro* assays, pSmad3 remained cytoplasmic in OvCa cells (supplementary material, Figure S3).

**Figure 5:**
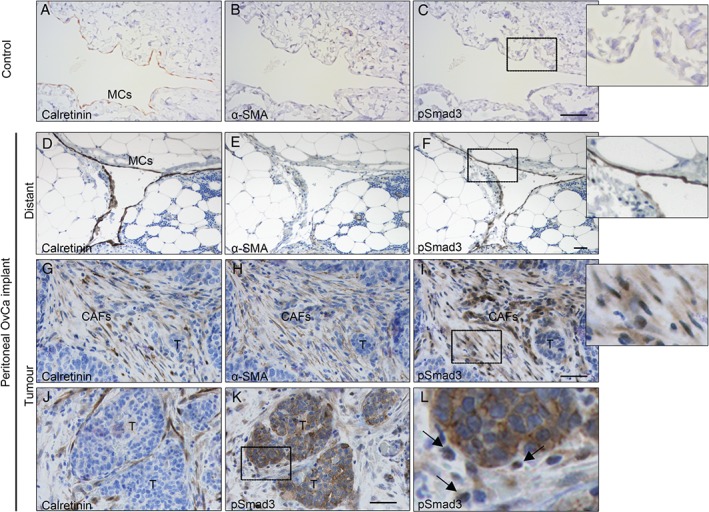
Immunohistochemical analysis of pSmad3 in human peritoneal implants of OvCa. Staining of serial sections was performed for calretinin, α‐SMA, and pSmad3. (A–C) The peritoneum of a control donor shows a preserved mesothelium (calretinin‐positive) that is negative for both α‐SMA and pSmad3 markers. (D–L) Same biopsy of an OvCa patient with peritoneal metastasis. (D–F) The preserved mesothelial monolayer (calretinin‐positive and α‐SMA‐negative) in an area distant from the tumour implant expresses nuclear pSmad3. (G–I) A submesothelial tumour implant shows surrounding stromal CAFs (α‐SMA‐positive) derived from MCs (calretinin‐positive) expressing nuclear pSmad3. (J, K) Micrometastasis area, showing OvCa cells with cytoplasmic expression of pSmad3, and stromal MCs (calretinin‐positive) expressing nuclear pSmad3. (L) Higher magnification of the delimited area in (K), where arrows point to cells with nuclear pSmad3 staining. Insets show higher magnification of the delimited areas in (C), (F) and (I), respectively. T, tumour. Scale bars: 50 µm.

The differential localization of pSmad3 in MC‐derived CAFs and cancer cells was also analysed by immunohistochemistry in serial sections of peritoneal implant biopsies from OvCa patients. The peritoneums of control donors showed a calretinin‐positive preserved mesothelial monolayer with no staining for α‐SMA or pSmad3 (Figure 5A–C). However, in OvCa patients, preserved mesothelial areas (calretinin‐positive) distant from tumour cells showed nuclear expression of pSmad3, indicating that the TGF‐β1 pathway had been activated (Figure 5D–F). Conversely, in the tumour stroma, several cells with spindle‐like morphology were triple‐positive for calretinin, α‐SMA, and nuclear pSmad3, confirming that MCs have activated TGF‐β–Smad3‐dependent signalling, undergone an MMT, invaded the stroma, and trans‐differentiated into CAFs (Figure 5G–I). Interestingly, OvCa peritoneal nodules from the same patient were pSmad3‐negative (Figure 5I) or showed pSmad3 staining limited to the cytoplasmic compartment (Figure 5J–L).

A similar localization pattern for pSmad3, nuclear in stromal MCs but not in the tumour, was also observed in peritoneal implants of colon cancer patients, suggesting that a crosstalk mechanism via TGF‐β1–pSmad3 could be common to cancers that metastasize via peritoneal carcinomatosis (supplementary material, Figure S4).

### Knockdown of Smad3 in the peritoneum reduces metastasis

**Figure 6:**
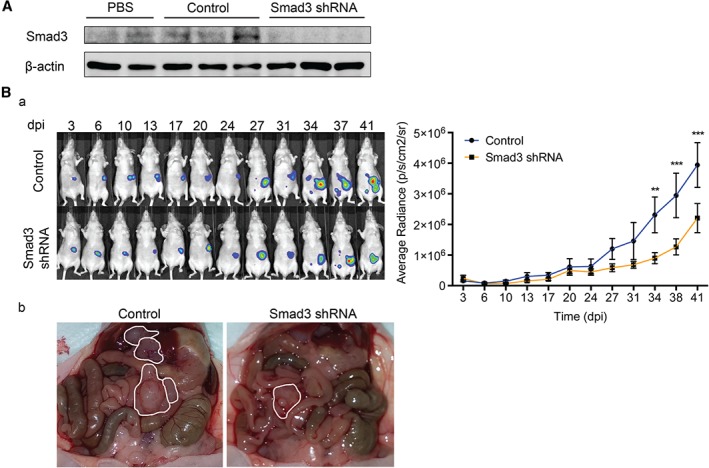
Lentiviral knockdown of Smad3 reduces tumour progression in the peritoneum. (A) Western blot shows the expression level of Smad3 in parietal peritoneum lysates of mice injected in a preliminary assay with PBS (n = 2), control lentiviral particles (n = 3), or Smad3 shRNA‐producing lentiviral particles (n = 3). Expression of β‐actin was employed as a loading control. (B) (a) Representative images of SKOV3‐luc‐D3 cell bioluminescence in mice pre‐conditioned with lentiviral particles producing Smad3 shRNA or control. Quantification of bioluminescence showed that tumour growth was significantly reduced in mice pre‐conditioned with lentiviral particles producing Smad3 shRNA (n = 9) as compared with controls (n = 9). Mice of both groups were monitored for 41 days. The graph represents mean average radiance (expressed as photons/s/cm^2^/sr) of SKOV3‐luc‐D3 cells ± standard error of the mean. Symbols represent the statistical differences over time between both groups (**p ≤ 0.01; ***p ≤ 0.001; p ≤ 0.0001). dpi, days post‐inoculation. (b) Representative images show a decrease in the number of metastases in Smad3 knockdown mice as compared with the control group. Tumours are outlined in white.

Once the relevance of the TGF‐β1–pSmad3 pathway in the communication between OvCa cells and HPMCs had been determined, lentiviral particles containing Smad3 shRNA were administered to mice in order to knock down its expression. In a preliminary assay, Smad3 knockdown was observable in the peritoneums of mice 4 days after inoculation of the lentivirus (Figure 6A). Then, lentiviral particles were administered intraperitoneally, and this was followed by inoculation of SKOV3‐luc‐D3 cells. Tumour progression was significantly reduced in mice whose peritoneal Smad3 expression had been knocked down as compared with controls (Figure 6Ba). Additionally, representative images showed that the number of intraperitoneal metastases was also reduced in mice in which Smad3 was silenced as compared with controls (Figure 6Bb).

## Discussion

Previous work from our group has shown that CAFs found in peritoneal metastatic implants from patients with abdominal cancer, such as ovarian, endometrial, or colorectal, derive from the mesothelium, through a tumour‐induced MMT 6. The mesenchymal conversion of MCs favours adhesion, invasion and growth by metastasizing cancer cells, through promoting profound structural modifications of the peritoneal niche, including matrix remodelling and angiogenesis 6, 7. Here, we demonstrate that MCs isolated from ascitic fluid of OvCa patients with peritoneal metastasis also undergo MMT. Moreover, as compared with control MCs, these trans‐differentiated AFMCs favour tumour progression in a subcutaneous xenograft mouse model, in which cancer cells alone did not grow. Tumour‐produced factors could continue to trans‐differentiate MCs, which would explain why control MCs also induced tumour growth, albeit less notably. Therefore, it is tempting to speculate that cancer cells depend on MC‐derived CAFs to progress through the peritoneum.

RNA sequencing provided a more detailed picture of the molecular differences, in a malignant context, between trans‐differentiated AFMCs and control MCs. The most differentially regulated pathway was hepatic stellate cell activation, which is in line with recent reports of MCs being converted into hepatic stellate cells and myofibroblasts through an MMT during liver injury 20. Other pathways are also relevant in the MMT/EMT and/or OvCa metastasis context, owing to: induction of MMT and peritoneal implantation through OvCa‐secreted molecules (TGF‐β and HGF) 6, 12; regulation of MMT/EMT or the TGF‐β1 pathway [integrin‐linked kinase (ILK), caveolin‐1, vitamin D receptor (VDR), thrombin, p38 mitogen‐activated protein kinase (MAPK), and nuclear factor‐κB (NF‐κB)] 21, 22, 23, 24, 25, 26, 27; or protection of the peritoneal membrane from the MMT (VDR and peroxisome proliferator‐activated receptor (PPAR) signalling) 28, 29. Interestingly, integrin signalling is also affected, supporting previous reports that, at initial stages of peritoneal metastasis, MMT enhances the binding of cancer cells to the peritoneum in a β1‐integrin‐dependent manner 6, 13. CAFs have been widely implicated in the remodelling of the ECM through MMPs, which are important molecular players in cancer progression by facilitating tumour invasion and vascularization 30, 31. Accordingly, MMP expression in OvCa has been linked to an increased metastatic potential 32. Additionally, there is an increase in MMP expression during *in vivo* MMT 33. Here, we show that AFMCs have high expression of VEGF, supporting previous reports describing MC‐derived CAFs as key players in tumour stroma vascularization 6. In this respect, MMP and angiogenesis pathways are differentially regulated in our RNA sequencing data, and pro‐angiogenic factors such as VEGF, fibroblast growth factor‐2, platelet‐derived growth factor, MMP1, MMP2, MMP3, MMP7 and MMP9 appear to be upregulated in trans‐differentiated AFMCs. Many soluble cytokines are present in high concentration in malignant OvCa ascitic fluid, including IL‐6, IL‐8, IL‐10, TGF‐β1, VEGF, IL‐1β, and TNF‐α 14, 15, 34, 35. On this note, IL‐6, IL‐8 and IL‐10 pathways are also differentially regulated in our RNA sequencing dataset and, interestingly, are associated with OvCa progression and poor prognosis 14, 34. The inflammatory cytokines IL‐1β and TNF‐α have been reported to work synergistically with TGF‐β1 in upregulating VEGF 36 and IL‐6 production in MCs 37. Supporting this, we show that TNF‐α, TGF‐β1, IL‐1β and IL‐6 are upstream regulators in our RNA sequencing data, which could point to MMT as a link between the inflammatory environment present in ascitic fluid and peritoneal metastasis.

We validated a five‐gene signature in biopsies of OvCa patients with peritoneal metastasis, observing that their protein products were also expressed in MCs (calretinin‐positive) with a fibroblastic phenotype surrounding the tumour implants. On this note, MMP1 has been proposed to induce chemokine production in OvCa cells, inducing angiogenesis 38. IL‐33 secreted by CAFs is involved in promoting EMT and invasion of head and neck squamous cancer cells 39. TSP1 has anti‐angiogenic properties 40; however, it also activates latent TGF‐β1 17, and its overexpression in gastric carcinoma CAFs is associated with tumour growth and nodal metastasis 41. GREM1 has been implicated in inducing MMT and angiogenesis by inhibiting bone morphogenetic protein (BMP)‐7 18, which has a protective role maintaining the epithelial phenotype of MCs 42. Finally, expression of EGR1 in stromal cells has been considered to be an independent prognostic indicator of poor survival in OvCa patients 43. Here, we demonstrate that the different pattern of expression observed in AFMCs reflects the changes that are taking place in stromal MCs in the peritoneum of OvCa patients. Further characterization of AFMCs in a larger number of patients – and correlation with cancer stage – could lead to a potential diagnostic and/or prognostic value of the gene signature proposed here, given that AFMCs are drained regularly by paracentesis.

TGF‐β1 is a key molecule driving MMT in different pathologies: peritoneal metastasis 6, fibrosis induced by peritoneal dialysis 9, and formation of postsurgical adhesions 44. We speculated that targeting the TGF‐β1 pathway could interfere with the accumulation of mesothelial‐derived CAFs, and therefore with tumour colonization through the peritoneum. Overexpression of TGF‐β1 in the peritoneum by adenoviral delivery indicated that it played a role in tumour progression. On the basis of previous results 6, we have blocked TGF‐β1 receptor I in a mouse model of peritoneal dissemination, demonstrating that OvCa‐induced MMT, at early stages, promotes a dramatic increase in tumour growth, whereas interfering with TGF‐β signalling reduces peritoneal metastasis. These results are supported by a previous report of *in vivo* TGF‐β1 blockade reducing peritoneal metastasis and improving survival in mice 45.

Here, we show that the Smad‐dependent pathway is activated in AFMCs, based on the nuclear localization of pSmad3, which is a necessary step for the subsequent transcriptional regulation 9. Interestingly, biopsies of patients with OvCa and colorectal peritoneal implants showed a differential localization of pSmad3 between cancer cells and the MC‐derived CAFs: cytoplasmic in the former, and nuclear in the latter. Our results suggest that, despite OvCa cells producing high amounts of TGF‐β1 6, paradoxically, the Smad‐dependent pathway appears to be disrupted. In fact, ovarian and colorectal tumours are characterized by a frequent loss of sensitivity to TGF‐β1, owing to mutations in its pathway 46, 47. On this note, OvCa cells have been reported to have lower levels of Smad4 48 or no Smad4 translocation into the nucleus 49. This could explain the cytoplasmic localization of pSmad3 in OvCa cells, given that Smad4 is necessary for this step 9. However, other studies have shown that OvCa cells respond to TGF‐β1 and undergo EMT with functional Smad signalling 50, 51. Therefore, further characterization of the Smad‐dependent and Smad‐independent pathways in both MCs and OvCa cells is needed to shed light on this differential regulation. Here, we have knocked down Smad3 in the peritoneums of mice, resulting in reduced intraperitoneal tumour growth and metastases. These data suggest that bidirectional communication between OvCa cells and MC‐derived CAFs, via TGF‐β–Smad‐dependent signalling, could be crucial to forming a suitable metastatic niche for peritoneal carcinomatosis.

In conclusion, we propose MMT as an alternative target in the treatment of metastases that disseminate via the peritoneum. Strategies that interfere with MMT in peritoneal dialysis could also be considered for the metastasis scenario, as the effects of MMT (MC invasion, fibroblast accumulation, ECM deposition, and angiogenesis) in the peritoneum seem to be similar in both pathologies 7. Further analysis of the pathways and molecules that are differentially regulated in the MCs isolated from OvCa patients could provide insights into novel mechanisms of peritoneal metastasis, as well as biomarkers for improving diagnosis and/or prognosis.

## Author contributions statement

The authors contributed in the following way: ARV, PS, MLC: designed research and developed the methodology; ARV, CLAU, MLPL, LCJ, TLY, PS: acquired and analysed experimental data; JAJH, CB, ICG, CFC: managed patients and provided surgical samples; SCM, PS, MLC: supervised the study; ARV, SCM, PS, MLC: wrote and reviewed the manuscript.

**SUPPLEMENTARY MATERIAL ONLINE**

**Supplementary materials and methods**

**Supplementary figure legends**

**Figure S1.** Ovarian carcinoma cells alone do not grow subcutaneously

**Figure S2.** Immunohistochemical marker validation in the mesothelium of ovarian cancer patients with peritoneal metastasis

**Figure S3.** Immunohistochemical analysis of pSmad3 in mouse peritoneal implants of ovarian cancer

**Figure S4.** Immunohistochemical analysis of pSmad3 in human peritoneal implants of colon cancer

**Table S1.** Specific primers for RT‐qPCR

**Table S2.** Top 100 upregulated genes in RNA‐seq data

**Table S3.** Top 100 downregulated genes in RNA‐seq data

**Table S4.** Upstream regulators in RNA‐seq data

## Supporting information


**Supplementary materials and methods**

**Supplementary figure legends**

**Figure S1 Ovarian carcinoma cells alone do not grow subcutaneously.** Representative bioluminescence images of in vivo monitoring of SKOV3‐luc‐D3 cells subcutaneously inoculated alone in left flank (orange circle) of mice and co‐inoculated with AFMCs in the right flank (blue circle) (n = 2). Mice were monitored for 29 days. Quantification of tumour‐emitted bioluminescence indicated that ovarian cancer cells alone do not grow subcutaneously. Graph represents mean average radiance (expressed as photons/s/cm2/sr) of SKOV3‐luc‐D3 cells ± SEM. dpi: days post‐inoculation.

**Figure S2 Immunohistochemical marker validation in the mesothelium of ovarian cancer patients with peritoneal metastasis.** The five‐gene signature chosen from the RNA sequencing dataset was analysed by immunohistochemistry in the mesothelial monolayer of peritoneal biopsies of advanced ovarian cancer patients. Serial sections from the same sample show superficial MCs (calretinin‐positive) overlapping with MMP1, EGR1 and GREM1 markers. A lower intensity of IL‐33 and TSP1 staining is found. Arrows indicate MCs that have started to invade through the submesothelial zone. Scale bars = 50 µm.

**Figure S3 Immunohistochemical analysis of pSmad3 in mouse peritoneal implants of ovarian cancer.** A, B. Peritoneum of a control mouse not harbouring cancer cells shows a preserved mesothelial monolayer, negative for α‐SMA and pSmad3. C‐H. Same sample from a mouse with ovarian cancer implants in the peritoneum. C, D. An area distant from the tumour implants shows a nuclear pSmad3‐positive mesothelium. E, F. Submesothelial CAFs expressing α‐SMA accumulate in an area close to the tumour site and overlap with nuclear pSmad3‐positive cells. G, H. Peritoneal tumour implant where ovarian cancer cells show cytoplasmic expression of pSmad3, and adjacent CAFs (α‐SMA‐positive) express nuclear pSmad3. Insets show higher magnifications of the delimited areas. Arrows indicate CAFs with nuclear pSmad3 staining in the proximity of tumour implants. T: Tumour. Scale bars = 50 µm. dpi: days post‐inoculation.

**Figure S4 Immunohistochemical analysis of pSmad3 in human peritoneal implants of colon cancer.** Staining of serial sections was performed for calretinin and pSmad3. A submesothelial colon cancer implant shows surrounding spindle‐like MCs (calretinin‐positive) expressing nuclear pSmad3. Tumour nuclei were negative for pSmad3. Insets show higher magnification of the delimited areas. Black arrows point to MCs with nuclear pSmad3 staining. White arrows indicate the lack of‐nuclear expression of pSmad3 in colon cancer cells. T: Tumour. S: Stroma. Scale bars: 50 µm.

**Table S1** Specific primers for qPCR.

**Table S2** Top 100 upregulated genes in RNA‐seq data.

**Table S3** Top 100 downregulated genes in RNA‐seq data.

**Table S4** Upstream regulators in RNA‐seq data.

## References

[1] Tan DS , Agarwal R , Kaye SB . Mechanisms of transcoelomic metastasis in ovarian cancer. Lancet Oncol 2006; 7: 925–934. 17081918 10.1016/S1470-2045(06)70939-1

[2] Della Pepa C , Tonini G , Pisano C . Ovarian cancer standard of care: are there real alternatives? Chin J Cancer 2015; 34: 17–27. 25556615 10.5732/cjc.014.10274 PMC4302086

[3] Hennessy BT , Coleman RL , Markman M . Ovarian cancer. Lancet 2009; 374: 1371–1382. 19793610 10.1016/S0140-6736(09)61338-6

[4] Siegel RL , Miller KD , Jemal A . Cancer statistics, 2016. CA Cancer J Clin 2016; 66: 7–30. 26742998 10.3322/caac.21332

[5] Di Paolo N , Sacchi G . Atlas of peritoneal histology. Perit Dial Int 2000; 20(suppl 3): S5–S96. 10877488

[6] Sandoval P , Jiménez‐Heffernan JA , Rynne‐Vidal A , *et al* Carcinoma‐associated fibroblasts derive from mesothelial cells via mesothelial‐to‐mesenchymal transition in peritoneal metastasis. J Pathol 2013; 231: 517–531. 24114721 10.1002/path.4281

[7] Rynne‐Vidal A , Jiménez‐Heffernan J , Fernández‐Chacón C , *et al* The mesothelial origin of carcinoma associated‐fibroblasts in peritoneal metastasis. Cancers 2015; 7: 1994–2011. 26426054 10.3390/cancers7040872 PMC4695872

[8] Yanez‐Mo M , Lara‐Pezzi E , Selgas R , *et al* Peritoneal dialysis and epithelial‐to‐mesenchymal transition of mesothelial cells. N Engl J Med 2003; 348: 403–413. 12556543 10.1056/NEJMoa020809

[9] López‐Cabrera M. Mesenchymal conversion of mesothelial cells is a key event in the pathophysiology of the peritoneum during peritoneal dialysis. Adv Med 2014; 2014: 1–17. 10.1155/2014/473134 PMC4590954 26556413

[10] Polanska UM , Orimo A . Carcinoma‐associated fibroblasts: non‐neoplastic tumour‐promoting mesenchymal cells. J Cell Physiol 2013; 228: 1651–1657. 23460038 10.1002/jcp.24347

[11] Mathot L , Stenninger J . Behavior of seeds and soil in the mechanism of metastasis: a deeper understanding. Cancer Sci 2012; 103: 626–631. 22212856 10.1111/j.1349-7006.2011.02195.x PMC7659190

[12] Nakamura M , Ono YJ , Kanemura M , *et al* Hepatocyte growth factor secreted by ovarian cancer cells stimulates peritoneal implantation via the mesothelial–mesenchymal transition of the peritoneum. Gynecol Oncol 2015; 139: 345–354. 26335595 10.1016/j.ygyno.2015.08.010

[13] Kenny HA , Chiang C‐Y , White EA , *et al* Mesothelial cells promote early ovarian cancer metastasis through fibronectin secretion. J Clin Invest 2014; 124: 4614–4628. 25202979 10.1172/JCI74778 PMC4191043

[14] Matte I , Lane D , Laplante C , *et al* Profiling of cytokines in human epithelial ovarian cancer ascites. Am J Cancer Res 2012; 2: 566–580. 22957308 PMC3433103

[15] Matte I , Lane D , Bachvarov D , *et al* Role of malignant ascites on human mesothelial cells and their gene expression profiles. BMC Cancer 2014; 14: 288. 24761768 10.1186/1471-2407-14-288 PMC4008408

[16] Loureiro J , Aguilera A , Selgas R , *et al* Blocking TGF‐β1 protects the peritoneal membrane from dialysate‐induced damage. J Am Soc Nephrol 2011; 22: 1682–1695. 21742730 10.1681/ASN.2010111197 PMC3171939

[17] Murphy‐Ullrich JE , Poczatek M . Activation of latent TGF‐beta by thrombospondin‐1: mechanisms and physiology. Cytokine Growth Factor Rev 2000; 11: 59–69. 10708953 10.1016/s1359-6101(99)00029-5

[18] Siddique I , Curran SP , Ghayur A , *et al* Gremlin promotes peritoneal membrane injury in an experimental mouse model and is associated with increased solute transport in peritoneal dialysis patients. Am J Pathol 2014; 184: 2976–2984. 25194662 10.1016/j.ajpath.2014.07.018 PMC5707198

[19] Patel P , Sekiguchi Y , Oh K‐H , *et al* Smad3‐dependent and ‐independent pathways are involved in peritoneal membrane injury. Kidney Int 2010; 77: 319–328. 19956083 10.1038/ki.2009.436

[20] Li Y , Wang J , Asahina K . Mesothelial cells give rise to hepatic stellate cells and myofibroblasts via mesothelial–mesenchymal transition in liver injury. Proc Natl Acad Sci U S A 2013; 110: 2324–2329. 23345421 10.1073/pnas.1214136110 PMC3568296

[21] Luo L , Liu H , Dong Z , *et al* Small interfering RNA targeting ILK inhibits EMT in human peritoneal mesothelial cells through phosphorylation of GSK‐3β. Mol Med Report 2014; 10: 137–144. 10.3892/mmr.2014.2162 24756461

[22] Vi L , de Lasa C , DiGuglielmo GM , *et al* Integrin‐linked kinase is required for TGF‐β1 induction of dermal myofibroblast differentiation. J Invest Dermatol 2010; 131: 586–593. 21150927 10.1038/jid.2010.362

[23] Razani B , Zhang XL , Bitzer M , *et al* Caveolin‐1 regulates transforming growth factor (TGF)‐beta/SMAD signaling through an interaction with the TGF‐beta type I receptor. J Biol Chem 2001; 276: 6727–6738. 11102446 10.1074/jbc.M008340200

[24] Strippoli R , Loureiro J , Moreno V , *et al* Caveolin‐1 deficiency induces a MEK‐ERK1/2‐Snail‐1‐dependent epithelial–mesenchymal transition and fibrosis during peritoneal dialysis. EMBO Mol Med 2015; 7: 102–123. 25550395 10.15252/emmm.201404127 PMC4309670

[25] Larriba MJ , de Herreros AG , Muñoz A . Vitamin D and the epithelial to mesenchymal transition. Stem Cells Int 2016; 2016: 1–12. 10.1155/2016/6213872 PMC4736588 26880977

[26] Zhong Y‐C , Zhang T , Di W , *et al* Thrombin promotes epithelial ovarian cancer cell invasion by inducing epithelial–mesenchymal transition. J Gynecol Oncol 2013; 24: 265–272. 23875077 10.3802/jgo.2013.24.3.265 PMC3714465

[27] Strippoli R , Benedicto I , Foronda M , *et al* p38 maintains E‐cadherin expression by modulating TAK1‐NF‐kappa B during epithelial‐to‐mesenchymal transition. J Cell Sci 2010; 123: 4321–4331. 21098640 10.1242/jcs.071647

[28] Sandoval P , Loureiro J , González‐Mateo G , *et al* PPAR‐γ agonist rosiglitazone protects peritoneal membrane from dialysis fluid‐induced damage. Lab Invest 2010; 90: 1517–1532. 20531289 10.1038/labinvest.2010.111

[29] González‐Mateo GT , Fernández‐Millara V , Bellón T , *et al* Paricalcitol reduces peritoneal fibrosis in mice through the activation of regulatory T cells and reduction in IL‐17 production. PLoS ONE 2014; 9: e108477. 10.1371/journal.pone.0108477 PMC4184804 25279459

[30] Paulsson J , Micke P . Prognostic relevance of cancer‐associated fibroblasts in human cancer. Semin Cancer Biol 2014; 25: 61–68. 24560651 10.1016/j.semcancer.2014.02.006

[31] Egeblad M , Werb Z . New functions for the matrix metalloproteinases in cancer progression. Nat Rev Cancer 2002; 2: 161–174. 11990853 10.1038/nrc745

[32] Al‐Alem L , Curry TEJ . Ovarian cancer: involvement of the matrix metalloproteinases. Reproduction 2015; 150: R55–R64. 25918438 10.1530/REP-14-0546 PMC4955511

[33] Margetts PJ , Bonniaud P , Liu L , *et al* Transient overexpression of TGF‐b1 induces epithelial mesenchymal transition in the rodent peritoneum. J Am Soc Nephrol 2005; 16: 425–436. 15590759 10.1681/ASN.2004060436

[34] Kolomeyevskaya N , Eng KH , Khan ANH , *et al* Cytokine profiling of ascites at primary surgery identifies an interaction of tumor necrosis factor‐α and interleukin‐6 in predicting reduced progression‐free survival in epithelial ovarian cancer. Gynecol Oncol 2015; 138: 352–357. 26001328 10.1016/j.ygyno.2015.05.009 PMC4522366

[35] Stadlmann S , Feichtinger H , Mikuz G , *et al* Interactions of human peritoneal mesothelial cells with serous ovarian cancer cell spheroids – evidence for a mechanical and paracrine barrier function of the peritoneal mesothelium. Int J Gynecol Cancer 2014; 24: 192–200. 24407573 10.1097/IGC.0000000000000036

[36] Catar R , Witowski J , Wagner P , *et al* The proto‐oncogene c‐Fos transcriptionally regulates VEGF production during peritoneal inflammation. Kidney Int 2013; 84: 1119–1128. 23760290 10.1038/ki.2013.217

[37] Offner FA , Obrist P , Stadlmann S , *et al* IL‐6 secretion by human peritoneal mesothelial and ovarian cancer cells. Cytokine 1995; 7: 542–547. 8580370 10.1006/cyto.1995.0073

[38] Agarwal A , Tressel SL , Kaimal R , *et al* Identification of a metalloprotease–chemokine signaling system in the ovarian cancer microenvironment: implications for antiangiogenic therapy. Cancer Res 2010; 70: 5880–5890. 20570895 10.1158/0008-5472.CAN-09-4341 PMC2917243

[39] Chen S‐F , Nieh S , Jao S‐W , *et al* The paracrine effect of cancer‐associated fibroblast‐induced interleukin‐33 regulates the invasiveness of head and neck squamous cell carcinoma. J Pathol 2013; 231: 180–189. 23775566 10.1002/path.4226

[40] Lawler PR , Lawler J . Molecular basis for the regulation of angiogenesis by thrombospondin‐1 and −2. Cold Spring Harb Perspect Med 2012; 2: a006627. 22553494 10.1101/cshperspect.a006627 PMC3331684

[41] Lin X‐D , Chen S‐Q , Qi Y‐L , *et al* Overexpression of thrombospondin‐1 in stromal myofibroblasts is associated with tumor growth and nodal metastasis in gastric carcinoma. J Surg Oncol 2012; 106: 94–100. 22231149 10.1002/jso.23037

[42] Loureiro J , Schilte M , Aguilera A , *et al* BMP‐7 blocks mesenchymal conversion of mesothelial cells and prevents peritoneal damage induced by dialysis fluid exposure. Nephrol Dial Transplant 2010; 25: 1098–1108. 20067910 10.1093/ndt/gfp618

[43] Kataoka F , Tsuda H , Arao T , *et al* EGRI and FOSB gene expressions in cancer stroma are independent prognostic indicators for epithelial ovarian cancer receiving standard therapy. Genes Chromosomes Cancer 2012; 51: 300–312. 22095904 10.1002/gcc.21916

[44] Sandoval P , Jiménez‐Heffernan JA , Guerra‐Azcona G , *et al* Mesothelial‐to‐mesenchymal transition in the pathogenesis of post‐surgical peritoneal adhesions. J Pathol 2016; 239: 48–59. 27071481 10.1002/path.4695

[45] Miao Z‐F , Zhao T‐T , Wang Z‐N , *et al* Transforming growth factor‐beta1 signaling blockade attenuates gastric cancer cell‐induced peritoneal mesothelial cell fibrosis and alleviates peritoneal dissemination both in vitro and in vivo. Tumour Biol 2013; 35: 3575–3583. 24347485 10.1007/s13277-013-1472-x

[46] Nilsson EE , Skinner MK . Role of transforming growth factor beta in ovarian surface epithelium biology and ovarian cancer. Reprod Biomed Online 2002; 5: 254–258. 12470522 10.1016/s1472-6483(10)61828-7

[47] Calon A , Espinet E , Palomo‐Ponce S , *et al* Dependency of colorectal cancer on a TGF‐β‐driven program in stromal cells for metastasis initiation. Cancer Cell 2012; 22: 571–584. 23153532 10.1016/j.ccr.2012.08.013 PMC3512565

[48] Antony ML , Nair R , Sebastian P , *et al* Changes in expression, and/or mutations in TGF‐beta receptors (TGF‐beta RI and TGF‐beta RII) and Smad 4 in human ovarian tumors. J Cancer Res Clin Oncol 2010; 136: 351–361. 19916025 10.1007/s00432-009-0703-4 PMC11828304

[49] Chan MW , Huang Y‐W , Hartman‐Frey C , *et al* Aberrant transforming growth factor beta1 signaling and SMAD4 nuclear translocation confer epigenetic repression of ADAM19 in ovarian cancer. Neoplasia 2008; 10: 908–919. 18714391 10.1593/neo.08540 PMC2517635

[50] Dunfield LD , Dwyer EJC , Nachtigal MW . TGF beta‐induced Smad signaling remains intact in primary human ovarian cancer cells. Endocrinology 2002; 143: 1174–1181. 11897669 10.1210/endo.143.4.8733

[51] Do T‐V , Kubba LA , Du H , *et al* Transforming growth factor‐beta1, transforming growth factor‐beta2, and transforming growth factor‐beta3 enhance ovarian cancer metastatic potential by inducing a Smad3‐dependent epithelial‐to‐mesenchymal transition. Mol Cancer Res 2008; 6: 695–705. 18505915 10.1158/1541-7786.MCR-07-0294 PMC2927222

