# Supplementary material for: Mesothelial‐to‐mesenchymal transition as a possible therapeutic target in peritoneal metastasis of ovarian cancer
Source: J Pathol. 2017 Apr 3;242(2):140–51. doi: 10.1002/path.4889 (PMC5468005; doi:10.1002/path.4889)
Supplement: Supplementary file 1 — Supplementary materials and methods [file PATH-242-140-s001.doc]

**SUPPLEMENTARY MATERIALS AND METHODS**

**RNA-sequencing, data analysis and functional annotations**

- Libraries were subjected to 100 cycles of paired-end (200 bp insert size) sequencing in the HiSeq 2000 (Illumina, San Diego, CA, USA), generating the following number of reads: 64,185,142 (HPMC 1); 55,795,924 (HPMC 2); 83,959,406 (HPMC 3); 72,360,862 (AFMC 1); 69,436,816 (AFMC 2); 66,494,626 (AFMC 3).
- Raw sequences obtained from the Illumina platform in FASTQ format were analysed using publicly available tools. Quality reads were analysed using FastQC. Due to the high quality of reads, there was no need to trim or filter them. The sequences were mapped to the GRCh38.p2 human reference genome sequence using a series of programs, including Bowtie2 for short-read mapping, and TopHat for defining exon–intron junctions. The expression level of each transcript was expressed as the fragments per transcript kilobase per million fragments mapped (FPKM) value, which was calculated based on the number of mapped reads using Cufflinks. Cuffdiff was used to detect differentially expressed genes using three replicates. Heatmaps were generated using heatmap.2 in the R package gplots (ver. 2.11.0) with the Z-scores of RPKM values.
- Genes that were differentially regulated were uploaded to the Ingenuity Pathway Analysis (IPA) tool (QIAGEN, Redwood City, CA, USA) to perform analysis of canonical pathways and upstream regulators. Fisher's exact test was used to measure the significance of the association between the list of genes and a canonical pathway. Pathways with a p-value < 0.05 (Benjamini-Hochberg method) were considered to be statistically significant. When analysing the resulting pathways in order to study their relevance in the context of ovarian cancer patients, canonical pathways that are specific to other cells types or unrelated pathologies were excluded. Upstream regulator analysis function was used to identify potential transcriptional regulators; and only the regulators that were also differentially expressed in our data set were considered. Upstream regulator analysis takes into account two measures: the statistical significance of the overlap between the genes in the dataset to and the list of downstream targets of a given regulator (p-value of overlap; *P* < 0.05 was considered significant), and the prediction of activation or inhibition of a putative regulator based on published findings included in the Ingenuity Knowledge Base (activation z-score; a z-score of ≥ |2| was considered significant).

***In vivo* bioluminescence imaging**

- In animal experiments where SKOV3-luc-D3 cells were inoculated: upon cervical injection of D-luciferin (Perkin-Elmer, Hopkinton, MA, USA) and anesthesia with inhaled isoflurane, luciferase signal was detected and tumour growth was monitored for 41 days (29 or 36 days in subcutaneous mouse model) with IVIS Lumina II (Perkin-Elmer). At the end of monitoring, animals were sacrificed upon CO_2_ inhalation and peritoneal tissue samples were recovered for further immunohistochemical analysis. Bioluminescent signal was quantified with Living Image 3.2 Software (Caliper Life Sciences, Hopkinton, MA, USA).

**Reverse Transcription – quantitative PCR (RT-qPCR) analysis**

MMT-related mRNAs were analysed by RT-qPCR in HPMCs and AFMCs. Cells were lysed in TRI Reagent (Ambion, Austin, TX, USA) and total RNA was extracted according to the manufacturer’s recommendations. Complementary DNA was obtained from 2 µg RNA by reverse transcription (Applied Biosystems, Cheshire, UK). Quantitative PCR was carried out in a LightCycler 480 II, using a SYBR Green kit (Roche Diagnostics, Barcelona, Spain) and specific primers for Snail, E-cadherin, VEGF-A and histone H3 (Supplementary Table S1). Samples were normalised with respect to the value obtained for H3.

**Immunofluorescence staining**

- HPMCs, AFMCs and SKOV3 cells were plated on 22 mm^2^ coverslips placed in 24-well tissue culture plates for immunofluorescence staining with antibodies to visualise calretinin (Polyclonal, 1:30, BioGenex, Fremont, CA, USA), α-smooth muscle actin (α-SMA) (Clone 1A4, 1:2000, Sigma-Aldrich, St. Louis, MI, USA) and pSmad3 (Clone C25A9, 1:100, Cell Signaling Technology, Inc. Danvers, MA, USA). Cells were fixed in 4% paraformaldehyde and permeabilised in 0.1% NP-40. In all cases, 5% donkey serum was applied to block non-specific unions. Secondary antibodies conjugated with Alexa 488, Alexa 555 or Alexa 647 (Thermo Fischer Scientific, Inc. Waltham, MA, USA) were incubated at room temperature. Nuclei were stained with 4,6-diamidino-2-phenylindole (DAPI) (Thermo Fisher Scientific) and images were captured with a Zeiss LSM710 confocal microscope (Zeiss, Oberkochen, Germany). Negative controls, in which primary antibodies were omitted, did not give rise to any detectable labelling. Where indicated, 50 nuclei were quantified using the analysis program Image-J 1.37c (National Institute of Health, Bethesda, Maryland).

**Immunohistochemical analysis**

- Human and mouse tissue samples were fixed in neutral-buffered 3.7% formalin and embedded in paraffin to immunohistochemical staining.
- Deparaffinised 3 µm serial sections from patient tissues were heated to expose the hidden antigens using Real Target Retrieval Solution containing citrate buffer, pH 6.0 (Dako, Glostrup, Denmark). Endogenous peroxidase was blocked with Real Peroxidase-Blocking Solution (Dako). Samples were stained using primary antibodies to detect Calretinin (Polyclonal, 1:200, Abcam, Cambridge, UK), α-SMA (Clone 1A4, 1:3000, Sigma Aldrich), pSmad3 (Clone C25A9, 1:300, Cell Signaling Technology), MMP1 (Clone 36665, 1:200, R&D Systems, Minneapolis, MN, USA), IL-33 (Clone 12B3C4, 1:1000, Merk Millipore, Billerica, MA, USA), EGR1 (Clone T.160.5, 1:100, Thermo Fisher Scientific), TSP1 (Clone A6.1, 1:100, LifeSpan BioSciences, Seattle, WA, USA) or GREM1 (Polyclonal, 1:300, Abgent, San Diego, CA, USA). Antibodies were visualised by means of a dextran–polymer conjugate technique (EnVision+, Dako) using 3,3´-diaminobenzidine (DAB) (Dako) as chromogen. Tissue sections were counterstained with haematoxylin.
- Similarly, deparaffinised mouse peritoneal samples were incubated with the following primary antibodies: E-Cadherin (Polyclonal, 1:800, Abcam), α-SMA (Clone 1A4, 1:3000, Sigma-Aldrich) or pSmad3 (Clone C25A9, 1:300, Cell Signaling Technology). Non-specific binding of secondary antibodies was blocked by pre-treating slides with goat serum. A biotinylated goat anti-rabbit IgG (H+L) was applied to detect rabbit primary antibodies, and complexes were visualised using the R.T.U Vectastain Elite ABC Kit (Vector Laboratories, Burlingame, CA, USA). Mouse primary antibodies were visualised applying the Vector M.O.M. Immunodetection Kit (Vector Laboratories) according to manufacturer’s instructions. Immunolabeling was visualised using DAB (Dako) as chromogen followed by haematoxylin counterstaining.

**Western blot**

- Mouse peritoneal tissues were lysed in RIPA buffer (1% sodium deoxycholate, 0.1% sodium dodecyl sulphate) plus a phosphatase and protease inhibitor cocktail (Thermo Fisher Scientific) and total protein was quantified with a protein assay kit (Thermo Fisher Scientific). Equal amounts of denatured protein (20 μg) from each sample were resolved by 7% sodium dodecyl sulphate–polyacrylamide gel electrophoresis under reducing conditions. Proteins were transferred onto nitrocellulose membranes, which were then blocked with 5% non-fat milk in TBS–Tween buffer for 1 h and incubated with a specific antibody against Smad2/3 (Clone 18, 1:1000, BD Biosciences, San Jose, CA, USA) in 0.5% milk in TBS-Tween overnight at 4 °C. The antibody was detected with a peroxidase-conjugated sheep anti-mouse antibody (GE Healthcare Life Science, Buckinghamshire, UK). The β-actin protein was detected with anti-β-actin-peroxidase conjugated immunoglobulin (Sigma-Aldrich). Finally, complexes were visualised with enhanced chemiluminescence detection kit (Thermo Fisher Scientific) and blot images were acquired using ImageQuant LAS 4000 Mini (GE Healthcare Life Science).

**Lentiviral production and titration**

- Plasmids containing the shRNA sequence targeting Smad3 or control were purchased in the pLKO.1-puro-CMV-TurboGFP™ backbone from Sigma-Aldrich. The MISSION® shRNA used was TRCN0000089026: 5’CCGGCTGTCCAATGTCA ACCGGAATCTCGAGATTCCGGTTGACATTGGACAGTTTTTG-3’. For bacterial transformation, 100 μl of competent bacteria were mixed with 10 ng of each plasmid and incubated on ice for 30 min. A 42 °C heat-shock was applied for 30 s, followed by immediate placement on ice for 5 min. SOC media (200 μl) was added to the bacterial suspensions before incubating at 37 °C for 1 h. Half of each the aliquot was plated out on 1.5% (w/v) LB agar plates with 100 μg/ml ampicillin at 37 °C overnight. The following day, single colonies were incubated in 200 ml of LB at 37 °C for 24 h. The LB medium was recollected and centrifuged at 4 ºC and 6,000 g for 20 min. The pellets obtained were used to purify the plasmid DNA with the QIAGEN Plasmid Maxi Kit (QIAGEN, Hilden, Germany) following manufacturer’s instructions. Pseudoviral particles were prepared using the pPACK‐F1 Lentivector packaging system (SBI System Biosciences, Mountain View, CA, USA) and transfected in HEK 293T producer cell line. The titration of pseudoviral particles generated with the lentiviral vectors was determined by calculating the percentage of positive GFP expression cells by flow cytometry in a BD FACSCalibur™ II flow cytometer (BD Biosciences) 72 h after infection and expressed in ifu/ml.

**Statistics**

- Statistical analyses were performed using GraphPad Prism version 5 (GraphPad Software, La Jolla, CA, USA). Results are represented as mean ± SEM in bar and line graphics; and as 25th and 75th percentiles, median, minimum and maximum values in box plots graphic of Figure 4. Data groups of Figures 1 and 4 were compared with the non-parametric Mann–Whitney rank sum U-test and *P* < 0.05 was considered statistically significant. Multiple t-test analysis was performed in Figures 1, 3 and 6, considering *P* < 0.01 statistically significant.
