# Supplementary figures and images for: Mesothelial‐to‐mesenchymal transition as a possible therapeutic target in peritoneal metastasis of ovarian cancer
Source: J Pathol. 2017 Apr 3;242(2):140–51. doi: 10.1002/path.4889 (PMC5468005; doi:10.1002/path.4889)

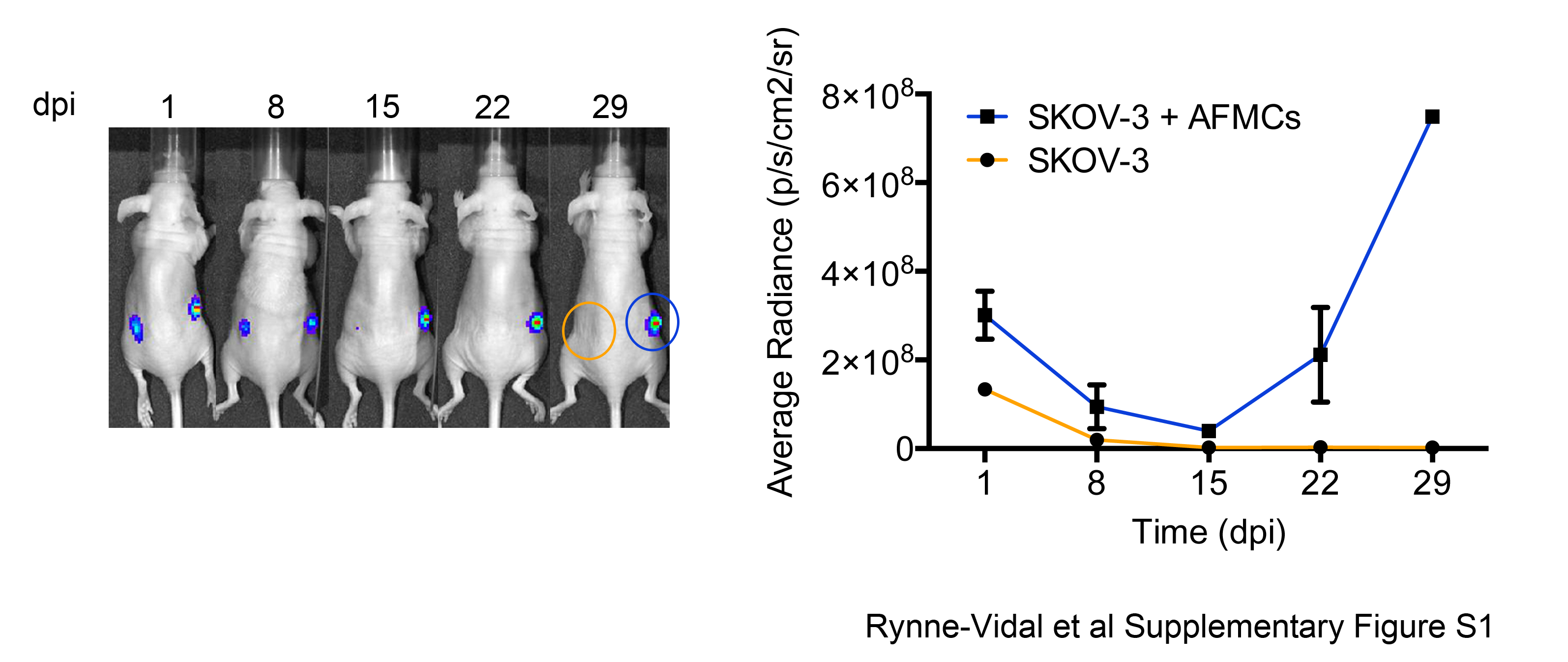

Supplement: Supplementary file 3 — Figure S1 Ovarian carcinoma cells alone do not grow subcutaneously. Representative bioluminescence images of in vivo monitoring of SKOV3‐luc‐D3 cells subcutaneously inoculated alone in left flank (orange circle) of mice and co‐inoculated with AFMCs in the right flank (blue circle) (n = 2). Mice were monitored for 29 days. Quantification of tumour‐emitted bioluminescence indicated that ovarian cancer cells alone do not grow subcutaneously. Graph represents mean average radiance (expressed as photons/s/cm2/sr) of SKOV3‐luc‐D3 cells ± SEM. dpi: days post‐inoculation. [file PATH-242-140-s003.tif]

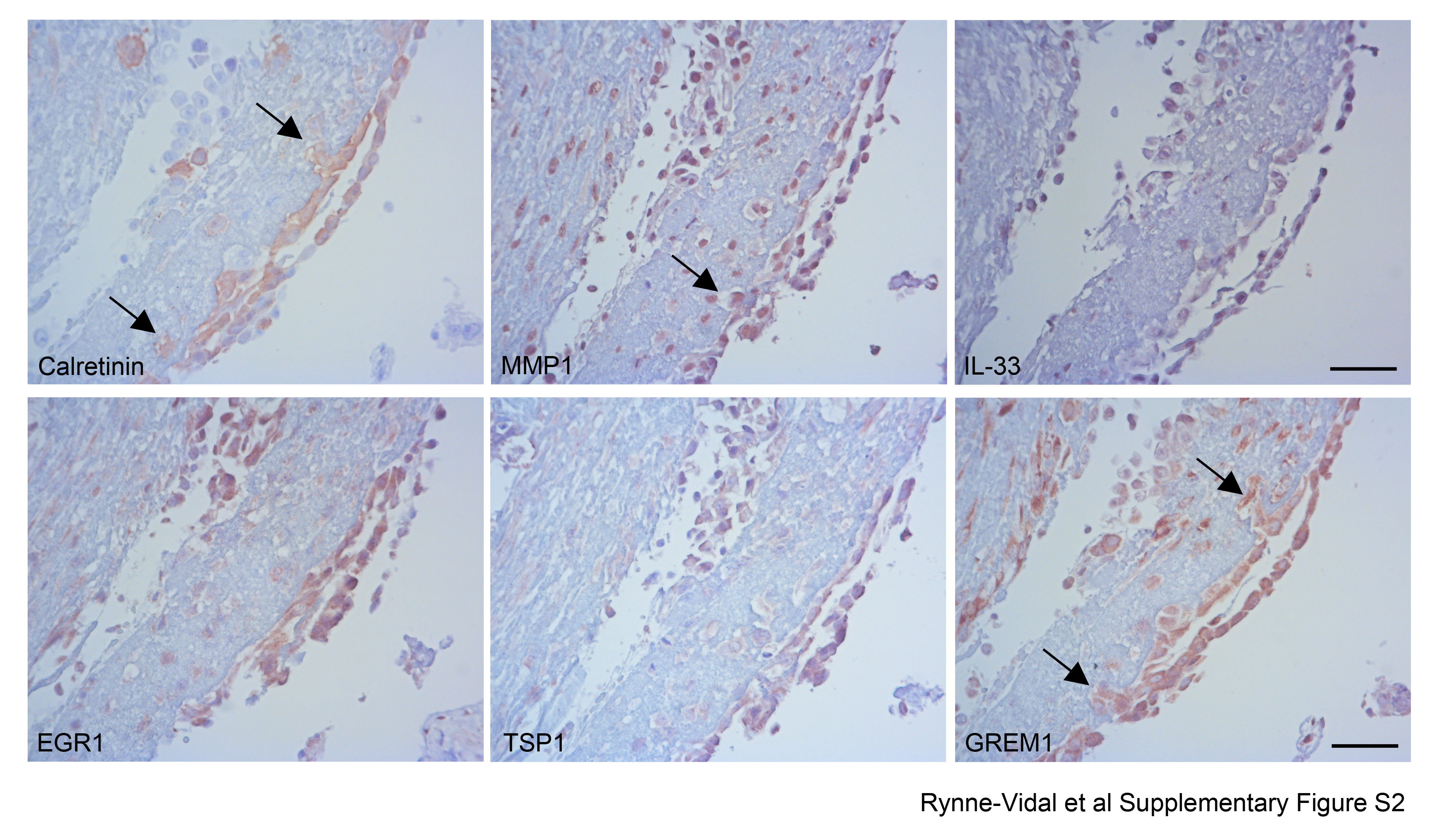

Supplement: Supplementary file 4 — Figure S2 Immunohistochemical marker validation in the mesothelium of ovarian cancer patients with peritoneal metastasis. The five‐gene signature chosen from the RNA sequencing dataset was analysed by immunohistochemistry in the mesothelial monolayer of peritoneal biopsies of advanced ovarian cancer patients. Serial sections from the same sample show superficial MCs (calretinin‐positive) overlapping with MMP1, EGR1 and GREM1 markers. A lower intensity of IL‐33 and TSP1 staining is found. Arrows indicate MCs that have started to invade through the submesothelial zone. Scale bars = 50 µm. [file PATH-242-140-s004.tif]

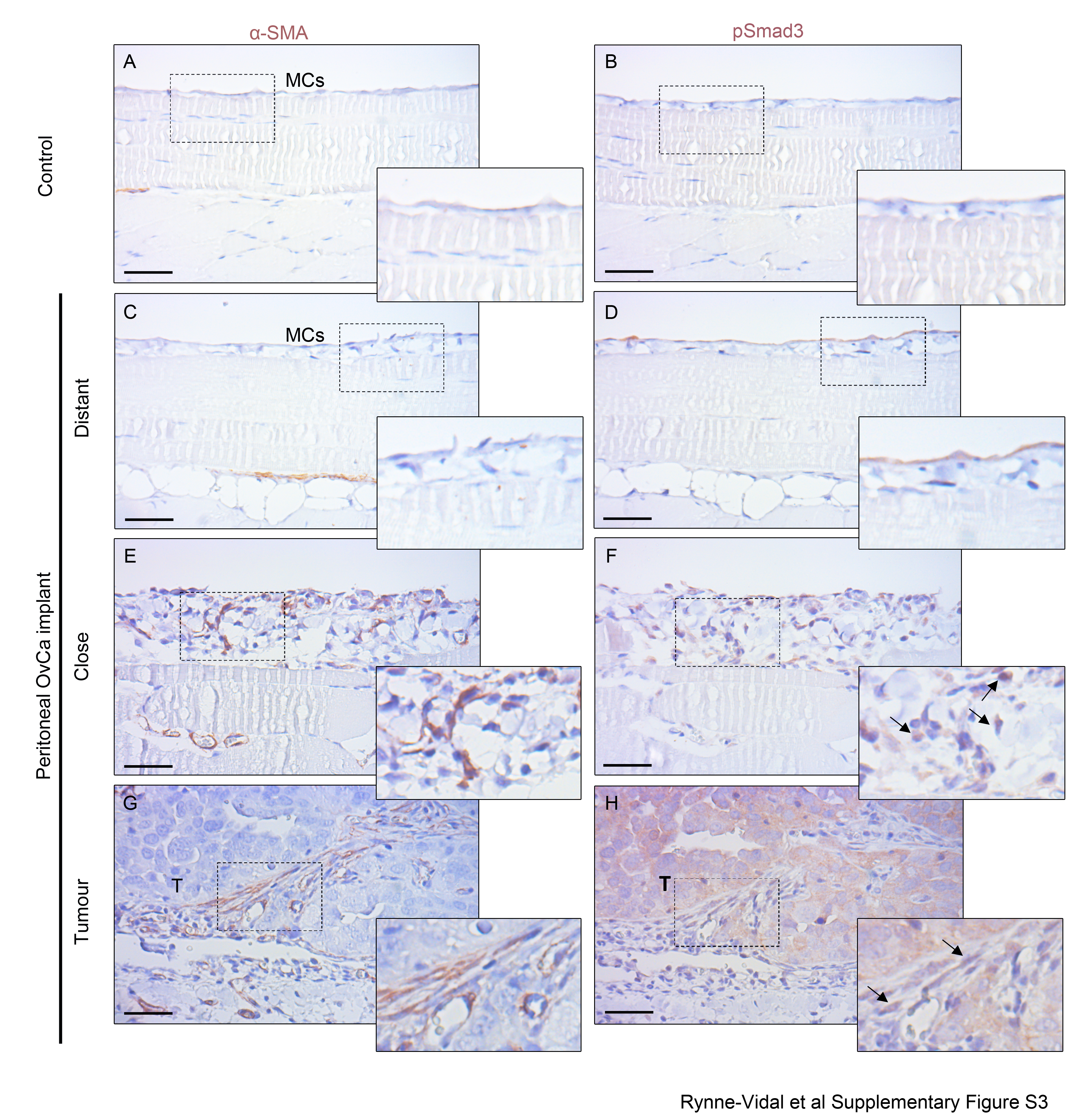

Supplement: Supplementary file 5 — Figure S3 Immunohistochemical analysis of pSmad3 in mouse peritoneal implants of ovarian cancer. A, B. Peritoneum of a control mouse not harbouring cancer cells shows a preserved mesothelial monolayer, negative for α‐SMA and pSmad3. C‐H. Same sample from a mouse with ovarian cancer implants in the peritoneum. C, D. An area distant from the tumour implants shows a nuclear pSmad3‐positive mesothelium. E, F. Submesothelial CAFs expressing α‐SMA accumulate in an area close to the tumour site and overlap with nuclear pSmad3‐positive cells. G, H. Peritoneal tumour implant where ovarian cancer cells show cytoplasmic expression of pSmad3, and adjacent CAFs (α‐SMA‐positive) express nuclear pSmad3. Insets show higher magnifications of the delimited areas. Arrows indicate CAFs with nuclear pSmad3 staining in the proximity of tumour implants. T: Tumour. Scale bars = 50 µm. dpi: days post‐inoculation. [file PATH-242-140-s005.tif]

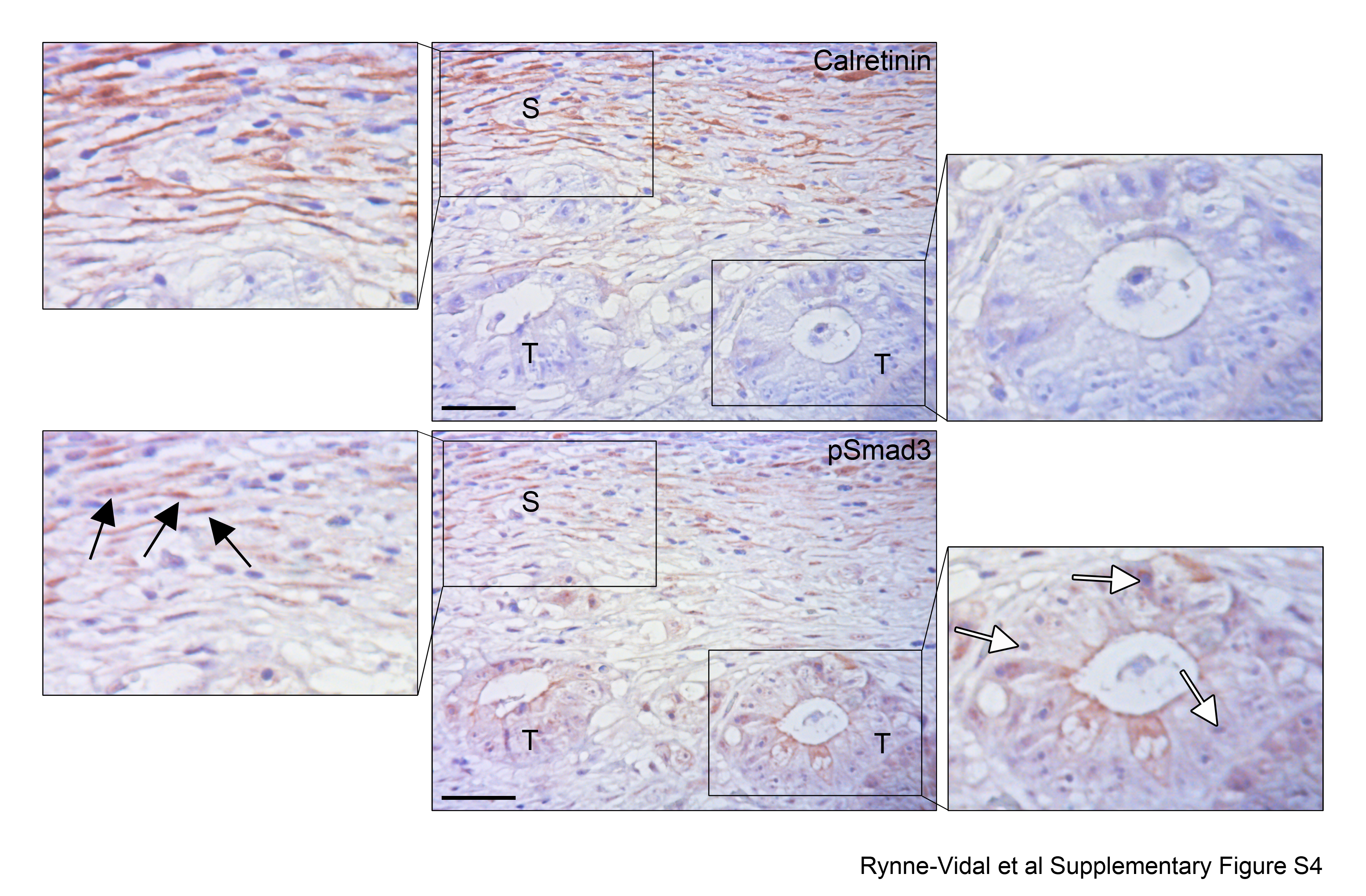

Supplement: Supplementary file 6 — Figure S4 Immunohistochemical analysis of pSmad3 in human peritoneal implants of colon cancer. Staining of serial sections was performed for calretinin and pSmad3. A submesothelial colon cancer implant shows surrounding spindle‐like MCs (calretinin‐positive) expressing nuclear pSmad3. Tumour nuclei were negative for pSmad3. Insets show higher magnification of the delimited areas. Black arrows point to MCs with nuclear pSmad3 staining. White arrows indicate the lack of‐nuclear expression of pSmad3 in colon cancer cells. T: Tumour. S: Stroma. Scale bars: 50 µm. [file PATH-242-140-s006.tif]
