# Supplementary material for: Mesothelial‐to‐mesenchymal transition as a possible therapeutic target in peritoneal metastasis of ovarian cancer
Source: J Pathol. 2017 Apr 3;242(2):140–51. doi: 10.1002/path.4889 (PMC5468005; doi:10.1002/path.4889)
Supplement: Supplementary file 7 — Table S1 Specific primers for qPCR. [file PATH-242-140-s007.docx]

**Table S1.** Specific primers for RT-qPCR.

| **Gene** | **Forward primer** | **Reverse primer** | ***T*_m_ (ºC)** |
| --- | --- | --- | --- |
| Snail | 5'-GCAAATACTGCAACAAGG-3' | 5'-GCACTGGTACTTCTTGACA-3' | 55 |
| E-cadherin | 5'-TGAAGGTGACAGAGCCTCTG-3' | 5'-TGGGTGAATTCGGGCTTGTT-3' | 62 |
| VEGF-A | 5'-GCAGAAGGAGGAGGGCAGAAT-3' | 5'-TATGTGCTGGCCTTGGTGAGG-3' | 60 |
| H3 Histone | 5'-AAAGCCGCTCGCAAGAGTGCG-3' | 5'-ACTTGCCTCCTGCAAAGCAC-3' | 62 |
