# Supplementary material for: Mesothelial‐to‐mesenchymal transition as a possible therapeutic target in peritoneal metastasis of ovarian cancer
Source: J Pathol. 2017 Apr 3;242(2):140–51. doi: 10.1002/path.4889 (PMC5468005; doi:10.1002/path.4889)
Supplement: Supplementary file 8 — Table S2 Top 100 upregulated genes in RNA‐seq data. [file PATH-242-140-s008.docx]

**Table S2.** Top 100 upregulated genes in RNA-seq data.

| **Symbol** | **Entrez Gene Name** | **Exp Log Ratio** | **Exp p-value** |
| --- | --- | --- | --- |
| MMP1 | matrix metallopeptidase 1 | 12.374 | 1.03E-01 |
| SIGLEC1 | sialic acid binding Ig-like lectin 1, sialoadhesin | 11.116 | 2.51E-01 |
| MMP3 | matrix metallopeptidase 3 | 10.298 | 7.00E-03 |
| HAMP | hepcidin antimicrobial peptide | 10.203 | 2.45E-01 |
| HK3 | hexokinase 3 (white cell) | 9.666 | 5.00E-05 |
| KLHDC7B | kelch domain containing 7B | 9.626 | 2.75E-03 |
| EHF | ets homologous factor | 9.601 | 2.29E-01 |
| CSF3 | colony stimulating factor 3 (granulocyte) | 9.412 | 3.40E-02 |
| ESM1 | endothelial cell-specific molecule 1 | 9.297 | 1.65E-03 |
| FPR3 | formyl peptide receptor 3 | 9.269 | 7.50E-04 |
| CCL4L1/CCL4L2 | chemokine (C-C motif) ligand 4-like 1 | 8.966 | 5.00E-05 |
| EGR3 | early growth response 3 | 8.943 | 1.00E-04 |
| STAB1 | stabilin 1 | 8.898 | 5.00E-05 |
| TREM1 | triggering receptor expressed on myeloid cells 1 | 8.889 | 1.34E-02 |
| NGEF | neuronal guanine nucleotide exchange factor | 8.771 | 5.00E-05 |
| NDUFA4L2 | NADH dehydrogenase (ubiquinone) 1 alpha subcomplex, 4-like 2 | 8.743 | 5.00E-05 |
| SERPINA3 | serpin peptidase inhibitor, clade A (alpha-1 antiproteinase, antitrypsin), member 3 | 8.704 | 4.11E-02 |
| SPP1 | secreted phosphoprotein 1 | 8.637 | 9.50E-04 |
| CXCL8 | chemokine (C-X-C motif) ligand 8 | 8.567 | 5.00E-05 |
| TNFAIP6 | tumor necrosis factor, alpha-induced protein 6 | 8.560 | 8.00E-04 |
| IL33 | interleukin 33 | 8.512 | 5.00E-05 |
| PTPN22 | protein tyrosine phosphatase, non-receptor type 22 (lymphoid) | 8.459 | 5.00E-05 |
| MME | membrane metallo-endopeptidase | 8.449 | 5.00E-05 |
| IFI44L | interferon-induced protein 44-like | 8.249 | 5.00E-05 |
| G0S2 | G0/G1 switch 2 | 8.200 | 5.10E-03 |
| CXCL3 | chemokine (C-X-C motif) ligand 3 | 8.101 | 1.10E-03 |
| IL1B | interleukin 1, beta | 8.091 | 5.00E-05 |
| NTM | neurotrimin | 8.080 | 5.45E-03 |
| WNT7A | wingless-type MMTV integration site family, member 7A | 8.063 | 1.00E00 |
| SPI1 | Spi-1 proto-oncogene | 8.037 | 8.82E-02 |
| SLC11A1 | solute carrier family 11 (proton-coupled divalent metal ion transporter), member 1 | 8.017 | 9.50E-04 |
| HSD11B1 | hydroxysteroid (11-beta) dehydrogenase 1 | 8.014 | 5.00E-05 |
| TNF | tumor necrosis factor | 7.971 | 4.50E-04 |
| LILRB2 | leukocyte immunoglobulin-like receptor, subfamily B (with TM and ITIM domains), member 2 | 7.924 | 5.00E-05 |
| TSLP | thymic stromal lymphopoietin | 7.924 | 2.00E-04 |
| MMP9 | matrix metallopeptidase 9 | 7.913 | 1.80E-03 |
| CSF2 | colony stimulating factor 2 (granulocyte-macrophage) | 7.900 | 1.18E-02 |
| PTX3 | pentraxin 3, long | 7.819 | 5.00E-05 |
| MSR1 | macrophage scavenger receptor 1 | 7.776 | 9.59E-02 |
| TREM2 | triggering receptor expressed on myeloid cells 2 | 7.709 | 2.42E-01 |
| HSPA7 | heat shock 70kDa protein 7 (HSP70B) | 7.613 | 1.37E-02 |
| KMO | kynurenine 3-monooxygenase (kynurenine 3-hydroxylase) | 7.611 | 5.00E-05 |
| LILRA6 | leukocyte immunoglobulin-like receptor, subfamily A (with TM domain), member 6 | 7.588 | 5.40E-03 |
| LYZ | lysozyme | 7.585 | 5.00E-05 |
| CLEC7A | C-type lectin domain family 7, member A | 7.561 | 5.60E-03 |
| CXCL1 | chemokine (C-X-C motif) ligand 1 (melanoma growth stimulating activity, alpha) | 7.393 | 5.00E-05 |
| LILRB4 | leukocyte immunoglobulin-like receptor, subfamily B (with TM and ITIM domains), member 4 | 7.329 | 2.31E-01 |
| GPR183 | G protein-coupled receptor 183 | 7.314 | 4.84E-02 |
| GNA15 | guanine nucleotide binding protein (G protein), alpha 15 (Gq class) | 7.269 | 1.00E00 |
| KYNU | kynureninase | 7.256 | 5.00E-05 |
| LILRB5 | leukocyte immunoglobulin-like receptor, subfamily B (with TM and ITIM domains), member 5 | 7.252 | 8.40E-03 |
| FOLR1 | folate receptor 1 (adult) | 7.210 | 2.39E-01 |
| MMP7 | matrix metallopeptidase 7 | 7.202 | 1.40E-01 |
| PTGER2 | prostaglandin E receptor 2 (subtype EP2), 53kDa | 7.197 | 1.00E00 |
| PTPRC | protein tyrosine phosphatase, receptor type, C | 7.188 | 6.45E-03 |
| COL6A3 | collagen, type VI, alpha 3 | 7.088 | 5.00E-05 |
| CCL3L3 | chemokine (C-C motif) ligand 3-like 3 | 7.054 | 5.00E-05 |
| TNFRSF6B | tumor necrosis factor receptor superfamily, member 6b, decoy | 7.031 | 1.00E00 |
| RSAD2 | radical S-adenosyl methionine domain containing 2 | 7.013 | 5.00E-05 |
| SYK | spleen tyrosine kinase | 6.949 | 6.09E-02 |
| GLI1 | GLI family zinc finger 1 | 6.896 | 6.00E-04 |
| CXCL2 | chemokine (C-X-C motif) ligand 2 | 6.860 | 5.00E-05 |
| FCGR3A/FCGR3B | Fc fragment of IgG, low affinity IIIa, receptor (CD16a) | 6.822 | 1.00E00 |
| CXCR4 | chemokine (C-X-C motif) receptor 4 | 6.753 | 2.19E-01 |
| ABCB4 | ATP-binding cassette, sub-family B (MDR/TAP), member 4 | 6.744 | 1.00E00 |
| HAS2 | hyaluronan synthase 2 | 6.735 | 2.09E-01 |
| SOX17 | SRY (sex determining region Y)-box 17 | 6.711 | 5.80E-03 |
| NCF4 | neutrophil cytosolic factor 4, 40kDa | 6.703 | 1.88E-01 |
| CXCL5 | chemokine (C-X-C motif) ligand 5 | 6.631 | 5.00E-05 |
| SLC44A4 | solute carrier family 44, member 4 | 6.604 | 1.12E-02 |
| GBP5 | guanylate binding protein 5 | 6.582 | 6.80E-03 |
| ANXA10 | annexin A10 | 6.578 | 5.00E-05 |
| INHBE | inhibin, beta E | 6.562 | 5.00E-05 |
| FCER1G | Fc fragment of IgE, high affinity I, receptor for; gamma polypeptide | 6.555 | 5.16E-02 |
| SLC7A11 | solute carrier family 7 (anionic amino acid transporter light chain, xc- system), member 11 | 6.514 | 5.00E-05 |
| SLA | Src-like-adaptor | 6.514 | 5.00E-05 |
| EGR1 | early growth response 1 | 6.462 | 5.00E-05 |
| FIBIN | fin bud initiation factor homolog (zebrafish) | 6.367 | 9.80E-03 |
| IL13RA2 | interleukin 13 receptor, alpha 2 | 6.365 | 5.00E-05 |
| CTHRC1 | collagen triple helix repeat containing 1 | 6.358 | 5.00E-05 |
| ADM2 | adrenomedullin 2 | 6.356 | 5.00E-05 |
| UCN2 | urocortin 2 | 6.347 | 1.00E00 |
| IRF8 | interferon regulatory factor 8 | 6.340 | 1.01E-01 |
| PHLDA1 | pleckstrin homology-like domain, family A, member 1 | 6.304 | 5.00E-05 |
| PTGS2 | prostaglandin-endoperoxide synthase 2 (prostaglandin G/H synthase and cyclooxygenase) | 6.302 | 5.00E-05 |
| XDH | xanthine dehydrogenase | 6.285 | 1.00E00 |
| HIF3A | hypoxia inducible factor 3, alpha subunit | 6.243 | 1.00E00 |
| FOSB | FBJ murine osteosarcoma viral oncogene homolog B | 6.223 | 5.00E-05 |
| KLRC4-KLRK1/KLRK1 | killer cell lectin-like receptor subfamily K, member 1 | 6.187 | 1.00E00 |
| CD52 | CD52 molecule | 6.184 | 1.56E-02 |
| RGCC | regulator of cell cycle | 6.179 | 2.41E-01 |
| CD69 | CD69 molecule | 6.173 | 1.00E00 |
| SLAMF8 | SLAM family member 8 | 6.169 | 5.00E-05 |
| FOXC2 | forkhead box C2 | 6.166 | 1.00E00 |
| ITGA11 | integrin, alpha 11 | 6.120 | 5.00E-05 |
| RPLP0P2 | ribosomal protein, large, P0 pseudogene 2 | 6.116 | 5.00E-05 |
| EPCAM | epithelial cell adhesion molecule | 6.038 | 5.00E-05 |
| C15orf48 | chromosome 15 open reading frame 48 | 5.996 | 5.00E-05 |
| KLF2 | Kruppel-like factor 2 | 5.989 | 5.00E-05 |
| C5AR1 | complement component 5a receptor 1 | 5.969 | 3.35E-03 |
