# Supplementary material for: Mesothelial‐to‐mesenchymal transition as a possible therapeutic target in peritoneal metastasis of ovarian cancer
Source: J Pathol. 2017 Apr 3;242(2):140–51. doi: 10.1002/path.4889 (PMC5468005; doi:10.1002/path.4889)
Supplement: Supplementary file 9 — Table S3 Top 100 downregulated genes in RNA‐seq data. [file PATH-242-140-s009.docx]

**Table S3.** Top 100 downregulated genes in RNA-seq data.

| **Symbol** | **Entrez Gene Name** | **Exp Log Ratio** | **Exp p-value** |
| --- | --- | --- | --- |
| CYP17A1 | cytochrome P450, family 17, subfamily A, polypeptide 1 | -11.305 | 1.65E-02 |
| SEL1L2 | sel-1 suppressor of lin-12-like 2 (C. elegans) | -8.976 | 5.00E-05 |
| SLC30A2 | solute carrier family 30 (zinc transporter), member 2 | -8.666 | 2.02E-02 |
| LRP2 | low density lipoprotein receptor-related protein 2 | -7.847 | 1.50E-04 |
| OVCH2 | ovochymase 2 (gene/pseudogene) | -7.681 | 5.00E-05 |
| SMTNL2 | smoothelin-like 2 | -7.581 | 9.50E-04 |
| NPHS1 | nephrosis 1, congenital, Finnish type (nephrin) | -7.570 | 5.00E-05 |
| SPANXN1 | SPANX family, member N1 | -7.432 | 1.00E-03 |
| MT1G | metallothionein 1G | -7.298 | 5.00E-05 |
| CAPN6 | calpain 6 | -7.162 | 1.00E-04 |
| ITM2A | integral membrane protein 2A | -7.072 | 5.00E-05 |
| KCNB1 | potassium channel, voltage gated Shab related subfamily B, member 1 | -6.979 | 5.00E-05 |
| RGS7BP | regulator of G-protein signaling 7 binding protein | -6.924 | 5.00E-05 |
| UPK3B | uroplakin 3B | -6.760 | 5.00E-05 |
| ITLN1 | intelectin 1 (galactofuranose binding) | -6.675 | 3.50E-04 |
| SPANXC | SPANX family, member C | -6.644 | 3.17E-02 |
| VIPR2 | vasoactive intestinal peptide receptor 2 | -6.546 | 5.00E-05 |
| CPB1 | carboxypeptidase B1 (tissue) | -6.525 | 5.00E-05 |
| SEPP1 | selenoprotein P, plasma, 1 | -6.443 | 5.00E-05 |
| KLK5 | kallikrein-related peptidase 5 | -6.367 | 2.00E-04 |
| MYCN | v-myc avian myelocytomatosis viral oncogene neuroblastoma derived homolog | -6.338 | 1.00E00 |
| ITIH3 | inter-alpha-trypsin inhibitor heavy chain 3 | -6.329 | 7.50E-04 |
| HBE1 | hemoglobin, epsilon 1 | -6.295 | 5.00E-05 |
| MT1H | metallothionein 1H | -6.096 | 3.75E-03 |
| OR51B4 | olfactory receptor, family 51, subfamily B, member 4 | -6.077 | 2.60E-02 |
| MYOZ2 | myozenin 2 | -5.648 | 5.00E-05 |
| KLK7 | kallikrein-related peptidase 7 | -5.594 | 2.00E-04 |
| KLHL31 | kelch-like family member 31 | -5.564 | 5.00E-05 |
| MTUS2 | microtubule associated tumor suppressor candidate 2 | -5.505 | 5.00E-05 |
| CLIC5 | chloride intracellular channel 5 | -5.498 | 5.00E-05 |
| MYL7 | myosin, light chain 7, regulatory | -5.478 | 1.15E-03 |
| SYNPO2L | synaptopodin 2-like | -5.403 | 5.00E-05 |
| SLC27A2 | solute carrier family 27 (fatty acid transporter), member 2 | -5.337 | 5.00E-05 |
| RGS5 | regulator of G-protein signaling 5 | -5.321 | 5.00E-05 |
| KCNA4 | potassium channel, voltage gated shaker related subfamily A, member 4 | -5.314 | 5.00E-05 |
| IGFL2 | IGF-like family member 2 | -5.235 | 5.00E-05 |
| FGF9 | fibroblast growth factor 9 | -5.220 | 1.90E-03 |
| NPR3 | natriuretic peptide receptor 3 | -5.202 | 5.00E-05 |
| FLT3 | fms-related tyrosine kinase 3 | -5.178 | 1.00E00 |
| ILDR2 | immunoglobulin-like domain containing receptor 2 | -5.172 | 5.00E-05 |
| SFTPD | surfactant protein D | -5.172 | 5.00E-05 |
| SERTM1 | serine-rich and transmembrane domain containing 1 | -5.155 | 5.00E-05 |
| SERPINE3 | serpin peptidase inhibitor, clade E (nexin, plasminogen activator inhibitor type 1), member 3 | -5.116 | 5.00E-05 |
| HPD | 4-hydroxyphenylpyruvate dioxygenase | -5.061 | 5.00E-05 |
| MRVI1 | murine retrovirus integration site 1 homolog | -5.050 | 5.00E-05 |
| CLSTN2 | calsyntenin 2 | -5.047 | 5.00E-05 |
| THBD | thrombomodulin | -5.038 | 5.00E-05 |
| TUBB4A | tubulin, beta 4A class IVa | -5.010 | 5.00E-05 |
| PRG4 | proteoglycan 4 | -4.970 | 4.50E-04 |
| IGFL3 | IGF-like family member 3 | -4.958 | 1.20E-03 |
| SBK2 | SH3 domain binding kinase family, member 2 | -4.917 | 9.70E-03 |
| CGN | cingulin | -4.891 | 5.00E-05 |
| WNT2B | wingless-type MMTV integration site family, member 2B | -4.889 | 5.00E-05 |
| HBD | hemoglobin, delta | -4.884 | 2.99E-02 |
| SMPD3 | sphingomyelin phosphodiesterase 3, neutral membrane (neutral sphingomyelinase II) | -4.841 | 5.00E-05 |
| HSD3B1 | hydroxy-delta-5-steroid dehydrogenase, 3 beta- and steroid delta-isomerase 1 | -4.840 | 6.50E-03 |
| CNGB1 | cyclic nucleotide gated channel beta 1 | -4.838 | 5.00E-05 |
| ERICH5 | glutamate-rich 5 | -4.832 | 5.00E-05 |
| KLK11 | kallikrein-related peptidase 11 | -4.809 | 5.00E-05 |
| ALOX15 | arachidonate 15-lipoxygenase | -4.798 | 1.35E-01 |
| NOX1 | NADPH oxidase 1 | -4.792 | 5.00E-05 |
| PTPRZ1 | protein tyrosine phosphatase, receptor-type, Z polypeptide 1 | -4.787 | 5.00E-05 |
| ZFP42 | ZFP42 zinc finger protein | -4.783 | 5.00E-04 |
| RSPO1 | R-spondin 1 | -4.770 | 5.00E-04 |
| GKN1 | gastrokine 1 | -4.754 | 9.50E-04 |
| PODXL | podocalyxin-like | -4.752 | 5.00E-05 |
| LAMB4 | laminin, beta 4 | -4.743 | 5.50E-04 |
| ANKRD2 | ankyrin repeat domain 2 (stretch responsive muscle) | -4.704 | 5.00E-05 |
| MYOZ1 | myozenin 1 | -4.692 | 2.15E-03 |
| MGAT4C | MGAT4 family, member C | -4.606 | 5.00E-05 |
| PIP5K1B | phosphatidylinositol-4-phosphate 5-kinase, type I, beta | -4.593 | 5.00E-05 |
| NRK | Nik related kinase | -4.578 | 5.00E-05 |
| MAOB | monoamine oxidase B | -4.569 | 5.00E-05 |
| ENPP1 | ectonucleotide pyrophosphatase/phosphodiesterase 1 | -4.565 | 5.00E-05 |
| CST6 | cystatin E/M | -4.536 | 5.00E-05 |
| CNTN5 | contactin 5 | -4.515 | 5.00E-05 |
| MUC19 | mucin 19, oligomeric | -4.461 | 6.75E-03 |
| CD244 | CD244 molecule, natural killer cell receptor 2B4 | -4.455 | 4.50E-04 |
| CTNNA2 | catenin (cadherin-associated protein), alpha 2 | -4.453 | 5.00E-05 |
| CPA4 | carboxypeptidase A4 | -4.410 | 5.00E-05 |
| HOGA1 | 4-hydroxy-2-oxoglutarate aldolase 1 | -4.407 | 5.00E-05 |
| CLDN15 | claudin 15 | -4.386 | 5.00E-05 |
| ITGA7 | integrin, alpha 7 | -4.386 | 5.00E-05 |
| KCNK2 | potassium channel, two pore domain subfamily K, member 2 | -4.379 | 5.00E-05 |
| SBSPON | somatomedin B and thrombospondin, type 1 domain containing | -4.350 | 5.00E-05 |
| PKHD1L1 | polycystic kidney and hepatic disease 1 (autosomal recessive)-like 1 | -4.317 | 5.00E-05 |
| MT1F | metallothionein 1F | -4.300 | 5.00E-05 |
| VTN | vitronectin | -4.268 | 5.00E-05 |
| CALB2 | calbindin 2 | -4.266 | 5.00E-05 |
| CYP24A1 | cytochrome P450, family 24, subfamily A, polypeptide 1 | -4.263 | 8.50E-04 |
| PSG9 | pregnancy specific beta-1-glycoprotein 9 | -4.223 | 5.00E-05 |
| SLPI | secretory leukocyte peptidase inhibitor | -4.212 | 5.00E-05 |
| REEP1 | receptor accessory protein 1 | -4.200 | 5.00E-05 |
| ISM2 | isthmin 2 | -4.175 | 5.00E-05 |
| HHIP | hedgehog interacting protein | -4.124 | 5.00E-05 |
| RAB17 | RAB17, member RAS oncogene family | -4.120 | 5.00E-05 |
| RAET1E | retinoic acid early transcript 1E | -4.110 | 5.00E-05 |
| EXTL1 | exostosin-like glycosyltransferase 1 | -4.092 | 1.05E-03 |
| FRY | furry homolog (Drosophila) | -4.069 | 5.00E-05 |
| DLGAP2 | discs, large (Drosophila) homolog-associated protein 2 | -4.055 | 5.00E-05 |
