# Supplementary material for: Mesothelial‐to‐mesenchymal transition as a possible therapeutic target in peritoneal metastasis of ovarian cancer
Source: J Pathol. 2017 Apr 3;242(2):140–51. doi: 10.1002/path.4889 (PMC5468005; doi:10.1002/path.4889)
Supplement: Supplementary file 10 — Table S4 Upstream regulators in RNA‐seq data. [file PATH-242-140-s010.docx]

**Table S4.** Upstream regulators in RNA-seq data.

| **Symbol** | **Gene name** | **Exp Log Ratio** | **Molecule Type** | **Predicted Activation State** | **Activation z-score** | **p-value of overlap** |
| --- | --- | --- | --- | --- | --- | --- |
| TNF | Tumor necrosis factor | 7.971 | cytokine | Activated | 9.536 | 3.93E-79 |
| TGFB1 | Transforming growth factor, beta 1 | 1.476 | growth factor | Activated | 5.952 | 3.47E-74 |
| IL1B | Interleukin 1 beta | 8.091 | cytokine | Activated | 8.121 | 1.69E-61 |
| HGF | Hepatocyte growth factor | 1.904 | growth factor | Activated | 6.527 | 3.04E-27 |
| IL6 | Interleukin 6 | 4.390 | cytokine | Activated | 5.511 | 6.73E-27 |
